# Clinical Insights on Caloric Restriction Mimetics for Mitigating Brain Aging and Related Neurodegeneration

**DOI:** 10.1007/s10571-024-01493-2

**Published:** 2024-10-16

**Authors:** Anchal Trisal, Abhishek Kumar Singh

**Affiliations:** 1 Department of Biosciences, Jamia Millia Islamia, New Delhi, 110 025 India; 2 Manipal Centre for Biotherapeutics Research, Manipal Academy of Higher Education, Karnatak Manipal, 576 104 India

**Keywords:** Aging, Caloric restriction, CR-mimetics, Clinical trials, Alzheimer’s disease, Parkinson’s disease

## Abstract

Aging, an inevitable physiological process leading to a progressive decline in bodily functions, has been an abundantly researched domain with studies attempting to slow it down and reduce its debilitating effects. Investigations into the cellular and molecular pathways associated with aging have allowed the formulation of therapeutic strategies. Of these, caloric restriction (CR) has been implicated for its role in promoting healthy aging by modulating key molecular targets like Insulin/IGF-1, mTOR, and sirtuins. However, CR requires dedication and commitment to a strict regimen which poses a difficulty in maintaining consistency. To maneuver around cumbersome diets, Caloric Restriction Mimetics (CRMs) have emerged as promising alternatives by mimicking the beneficial effects of CR. This review elucidates the molecular foundations enabling CRMs like rapamycin, metformin, resveratrol, spermidine, and many more to function as suitable anti-aging molecules. Moreover, it explores clinical trials (retrieved from the clinicaltrials.gov database) aimed at demonstrating the efficacy of CRMs as effective candidates against age-related neurodegenerative diseases such as Alzheimer’s disease and Parkinson’s disease.

## Introduction

Characterized by declining health and loss of bodily functions, aging is a natural phenomenon resulting from a complex interplay between changing cellular and molecular pathways that coalesce to make the individual more susceptible to life-threatening conditions and diseases (Bhaduri et al. 2023). Age-associated neurodegeneration is amongst the most debilitating conditions that have been increasing in prevalence among the elderly in the last decade. Neurodegenerative disorders (NDDs) like Alzheimer’s disease, Parkinson’s disease, and Huntington’s disease have a grave impact on the quality of life of the people affected, leading them to depend on external help for everyday tasks (Mathur et al. 2023). Typified by declining cognition and reduced physical ability, these debilitating disorders lack early-onset interventions that can mitigate the extent of disease progression. As an effort to address this situation, researchers have looked upon nutritional manipulation as a potential therapeutic intervention to alleviate some of the duress associated with these diseases (Rahman et al. 2020). Studies have shown that modulating diet to incorporate beneficial nutrients has helped in the alleviation of symptoms associated with NDDs and in general promoted “healthy aging” (Milošević et al. 2021).

**Fig. 1:**
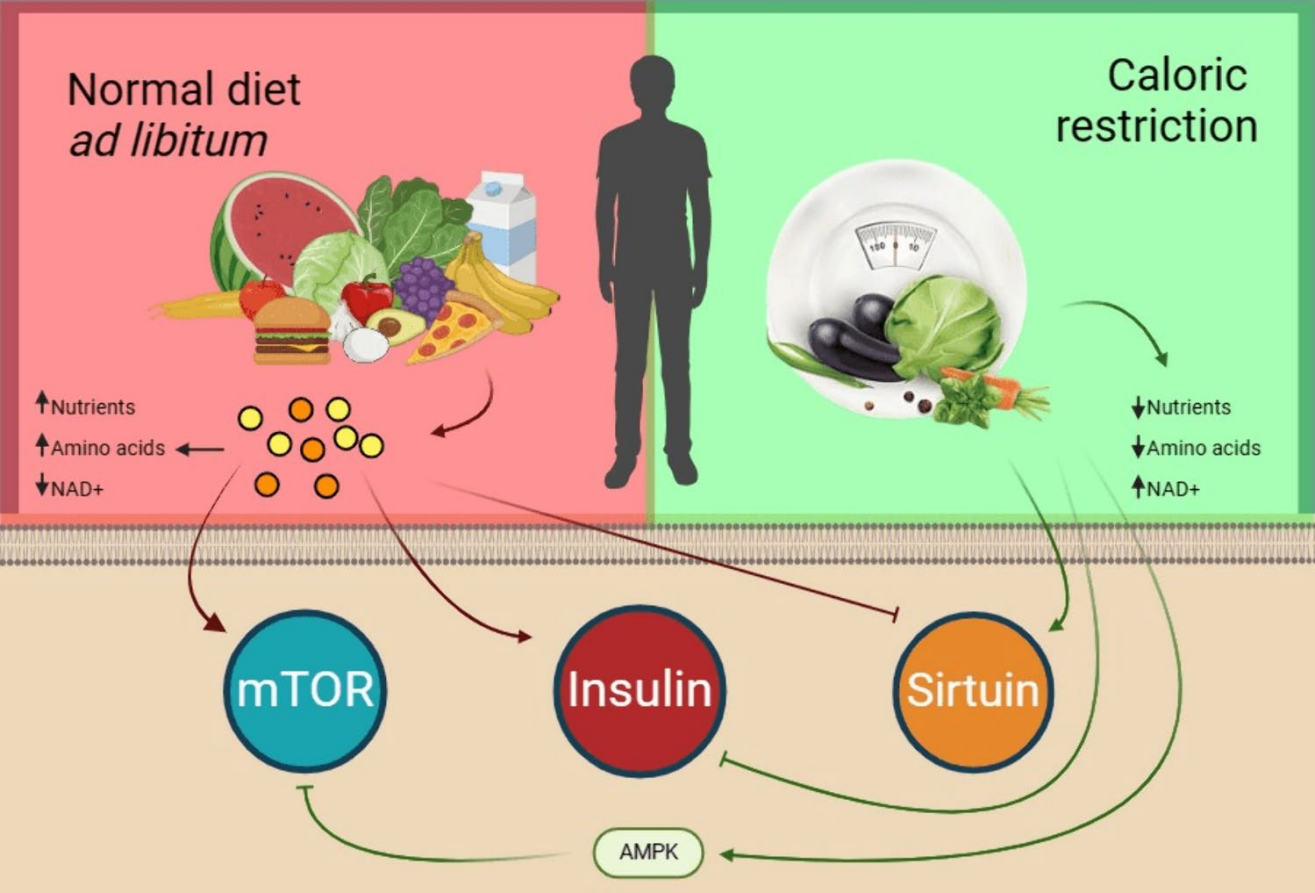
An illustration highlighting the effect of normal diet vs. caloric restriction on major nutrient sensing pathways; mTOR, Insulin/IGF-1, and Sirtuins

Caloric restriction (CR) is a dietary routine that is responsible for reducing the energy derived from the macro and micronutrients available in foods while not incurring malnutrition. It is a natural therapy that aims at increasing longevity, reducing inflammatory markers like cytokines and neurotrophic factors, and reducing the extent of oxidative damage (Kökten et al. 2021). A 20–30% reduction in calorie intake has been proven sufficient to elucidate beneficial effects (Redman and Ravussin 2011). With various studies having been conducted over the past decade, there is a consensus amongst the scientific community that caloric restriction is the most effective way to maximize longevity (Redman and Ravussin 2011; Dakic et al. 2022). Moreover, studies have shown that CR has had an influence on the onset of age-associated diseases and helped in mitigating the adverse effects associated with them (Kim et al. 2020). In other words, caloric restriction improves the health span of the affected individual, i.e., the period of life not affected by a fatal disease. The percentage reduction amongst humans and other species varies, with most rodent models requiring a higher, 30–40% reduction in calories for beneficial effects to show. Therefore, translational research in this domain is still underway for the accurate percentage of reduction which can be beneficial to humans. CR was first identified for its anti-aging effects by Lane et al. (1998). The mechanism behind CR-based increase in longevity has still not been completely elucidated but there is evidence suggesting that CR has an impact on molecular pathways that govern cellular aging processes and the advent of senescence-associated phenotypes. These include the insulin pathway, TOR pathway, and the role of Sirtuins. It has been speculated that an interplay of these pathways and their modulation is the reason behind the beneficial effects associated with CR (Mayor 2023) (Fig. 1). However, in a practical scenario, upholding a strict regimen is not feasible. To combat this, gerontologists have devised certain molecules, derived from natural compounds that mimic these effects associated with CR (Kim et al. 2020). They are termed caloric restriction mimetics (CRMs) and work by modulating the same pathways involved in CR without the hassle of having to adhere to a strict diet (Hofer et al. 2021).

CRMs are pharmacologically active substances that mimic the effect of CR. CRMs were first identified by Ingram et al. for their emergence as an alternative to CR (Ingram et al. 2006; Ingram and Roth 2011). For a compound to be characterized as a CRM, it should meet certain criteria such as they should increase lifespan, decrease the extent of age-associated disorders, induce autophagy, and reduce protein acetylation (Hofer et al. 2021). The mechanism by which each of these effects is achieved varies among different CRM candidates. These candidates may include natural plant-based compounds or synthetic compounds manufactured in laboratories (Nishizono et al. 2017). CRMs usually exhibit pleiotropic effects with cascading effects on many different target proteins. This poses an advantage in holistically improving health-span (Liu 2022). Most of the CRMs are either natural compounds safe to consume or FDA-approved drugs which makes their candidacy as therapeutic molecules against NDDs more appealing and approachable. There is an abundance in the number of in-vivo studies establishing the role of CRMs as viable candidates for mitigating the severity of NDDs which have further enabled the establishment of clinical investigations that seek to reproduce the results obtained from in-vivo studies in human subjects. Several clinical studies have been conducted which explore the putative role of CRMs in mitigating the effects of NDDs. In this review, we aim to summarize all completed and ongoing clinical trials on CR and CRMs and give an overview of their reported results, outcome measures, and caveats.

## A Brief Overview of Age-Associated Phenotypes and Neurodegenerative Disorders

Specific phenotypic characteristics emerge with increasing age. Alongside a decline in physical capability and strength (characterized by decreased grip strength, aberrant gait, imbalance, lack of endurance, and reduced dexterity), we also observe physiological and metabolic disturbances altered insulin sensitization, and mitochondrial dysfunction leading to an accumulation of reactive oxygen species (ROS) (Boemi et al. 2016; Bhaduri et al. 2023). ROS can further contribute to a decline in cellular integrity in the way of protein misfolding, lipid peroxidation, and formation of glycated end-products (Bhaduri et al. 2023). Another major metabolic dysfunction observed with increasing age is the decline in autophagy and increasing abundance of autophagic vesicles, resulting in the accumulation of cellular toxicants. Chronic systemic inflammation also accompanies aging; a phenomenon termed as “inflammaging”. It is characterized by an inflated level of inflammatory cytokines like IL-6, TNF-α, and IL-1β along with decreased functionality of lymphoid organs (Li et al. 2023a, b). Cognitive dysfunction is a consequence of cerebral aging with an evident effect on memory storage and retrieval and an axiomatic impediment on executive functions resulting in loss in the ability to conduct menial tasks (Brito et al. 2023).

### Pathophysiology of Alzheimer’s Disease and Parkinson’s Disease

NDDs, viz. Alzheimer’s disease (AD) and Parkinson’s disease (PD), have been a formidable thorn in humans for centuries. AD, which is typified by neurofibrillary tangles (NFTs) and the aggregation of senile plaques that are formed by the amalgamation of amyloid-β protein, a truncated product of APP (Amyloid precursor protein) cleaved by the enzyme β-secretase (BACE1), is the foremost contributor to NDDs (Xie et al. 2014). Aggregation of amyloid-β protein is a significant contributor to the onset of vascular damage, oxidative stress, and the genesis of NFTs (Neurofibrillary tangles) by triggering a downstream signalling cascade. Oxidative stress-mediated neuronal death is another facet of AD, leading to functional deficits like memory loss (Zhang et al. 2018). Starting from the hippocampus and later affecting the cortex, neuronal damage causes impediments in language, reasoning, and behaviour (Anand and Dhikav 2012). More significantly, a loss in cholinergic systems is observed, which has led to the establishment of acetylcholinesterase (AChE) as an important target for drug discovery (Ferreira-Vieira et al. 2016).

Parkinson’s disease (PD), which is exemplified by dyskinesia and often potentiates cognitive perturbations, is second only to AD in terms of the prevalence of NDDs worldwide. The assemblage of the neuronal protein α-synuclein in the form of Lewy bodies in dopaminergic neurons of substantia nigra has long been thought of to be the pivotal pathophysiological mechanism behind PD (Venda et al. 2010). Dopamine has also been purported to be a causative agent behind the oxidization of α-synuclein. Literature on the pivotal role of oxidative stress in the onset of PD is abundant. ROS-mediated aggregation of α-synuclein has been reported to be linked intrinsically to the biological process of aging and the coalescence of aging factors within the cytosol. This is exacerbated even further under certain physiological conditions, viz. mitochondrial stress which heightens the intracellular accumulation of α-synuclein(Bhaduri et al. 2023). Owing to the progressive degeneration of dopaminergic neurons in the substantia nigra in PD, dopaminergic neurotransmission in the corpus striatum is greatly reduced (Ramesh and Arachchige 2023). Clinical manifestations of PD such as restricted movement owing to rigidity, bradykinesia, and tremors lead to the worsening of the quality of life in PD-affected patients (Kouli et al. 2018).

## Pathways Involved in CR and CRM-Based Therapeutics

The major pathways involved in elucidating the therapeutic effect of CR and CRMs include the insulin pathway, mTOR/AMPK pathway, and the molecular interactions of sirtuins.

A serine-threonine receptor belonging to the PI3K kinase family, mTOR is a nutrient-sensing molecule that is sensitive to amino acid concentration. It plays a role in mediating diet-induced senescence. mTOR complexes are activated by various pathways including PI3K/AKT pathway or via small GTPases (Sharma and Singh 2023). mTORC1 promotes anabolic processes like proteostasis and lipid biosynthesis while inhibiting autophagy (Dossou and Basu 2019). There is a crosstalk between mTOR and AMPK. More specifically, AMPK, via activation of downstream regulators, inhibits mTOR complexes. AMPK also possesses the ability to directly phosphorylate and inactivate members of the mTOR complex. Along with the phosphorylation of ULK1, this is a major way in which AMPK induces autophagy (Garza-Lombó et al. 2018). Based on the energy level of the body, AMPK detects the status and signals for the activation of mTORC1. mTORC1 further activates transcription factors associated with mRNA translation (S6K1, eIF4F), glucose and lipid metabolism (SREBP, H1F1α), and inhibited autophagy via inactivation of ULK1 and ATG14 genes (Saxton and Sabatini 2017). mTORC2 plays an important role in cell survival and proliferation. It modulates the expression of the FoxO group of transcription factors and inhibits apoptosis while allowing the cell to progress in the cell cycle (Laplante and Sabatini 2009). Increased mTOR signaling is one of the cellular markers of aging and manifests in most age-related disorders. mTOR pathway inhibitors therefore act as CRMs to induce autophagy and promote healthy aging (Ham et al. 2022).

CRMs modulate insulin signaling by decreasing the systemic levels of insulin/IGF (Lee and Min 2013). Essential for cell proliferation and survival, this pathway controls several cellular functions like apoptosis, autophagy, and expression of immunogenic factors. Activation of this pathway causes the inhibition of the pro-apoptotic protein BAD and promotes protein synthesis (Iams and Lovly 2015). With apoptotic processes on a halt, increased activation of the insulin-dependent PI3K pathway leads to the accumulation of damaged cells within the body. Impaired apoptosis also enhances the accumulation of damaged proteins. In aged cells, the accumulation of lipid peroxides, reactive oxygen species, and glycation end products is higher due to impaired mitochondrial activity. This causes modification in proteins that can be detrimental (Bhatti et al. 2022). Reduced insulin signaling promotes apoptosis and autophagy and therefore plays a crucial role in the clearance of toxic biomolecules. Low levels of insulin and IGF1 promote the expression of FoxO transcription factors which promote healthy progression of the cell cycle (Link and Fernandez-Marcos 2017; Shintani et al. 2018).

**Fig. 2:**
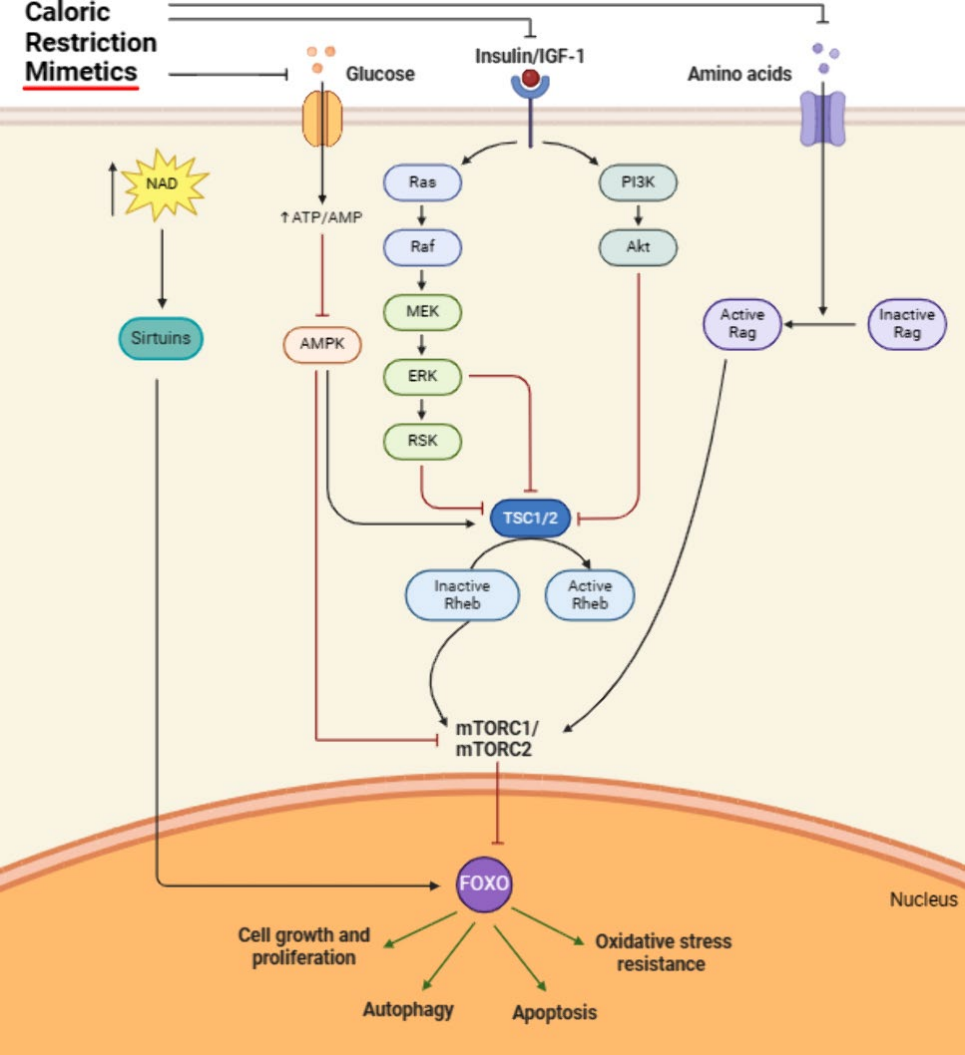
An illustration elucidating the downstream molecular players in nutrient sensing pathways, their cellular effects, and their regulation by Caloric Restriction Mimetics

A family of histone deacetylases, sirtuins, play an important role in regulating aging. They respond to cellular changes like DNA damage and help in maintaining cellular homeostasis. Sirt1 decreases the expression of p16, a protein involved in cell cycle arrest, thereby preventing cellular senescence (Rayess et al. 2012). It also suppresses NF-κB, an inflammatory molecule implicated in several neurodegenerative diseases. Sirt3 is an important molecule for increased longevity. Activated Sirt3 results in optimal oxygen utilization thereby reducing the generation of ROS. Another study corroborated that Sirt6 downregulates the IGF signaling pathway, leading to healthy aging (Bhaduri et al. 2023). In addition to this, sirtuins also act as regulators of autophagy. They deacetylate various Atg genes associated with the initiation of autophagy. Indirectly, sirtuins deacetylate FOXO transcription factors that are further involved in the expression of proteins of autophagic processes (Lee 2019). The pathways have been summarized in Fig. 2.

## Benefits of CR in Clinical Practice: Analysis of Clinical Trials

One of the leading attestations to the beneficial effects of CR comes from a rigorous study in humans termed CALERIE (Comprehensive Assessment of Long-Term Effects of Reducing Intake of Energy) organized by the National Institute of Aging. With a span of over 9 years, the study comprised three branches, with two pilot trials lasting 6–12 months each and a large multisite randomized study termed CALERIE 2. The first two pilot studies served as a preliminary base for formulating the design for the 25% caloric restriction adopted in CALERIE 2 (Rickman et al. 2011). The study aimed to reduce the caloric intake of 220 + participants without imposing a strict nutrient chart. Three diet options were presented including low-fat foods, low glycaemic content foods, and a Mediterranean diet.

The CALERIE 2 intervention aimed to reduce caloric intake without enforcing a set nutrient composition and required only that the self-selected diets meet daily micronutrient requirements. The participants were kept on a schedule for the first month of the trial to help them gauge their nutrient intake and manipulate their diet according to their preferences while still maintaining a calorie deficit. All diet options presented had a fiber ratio of 14 g per 1000 kcal and the nutrient composition varied between 15 and 30% protein, 40% and 60% carbohydrate, and 20% and 35% fat. The three diets were low fat, low glycemic load, and Mediterranean diets.

**Fig. 3:**
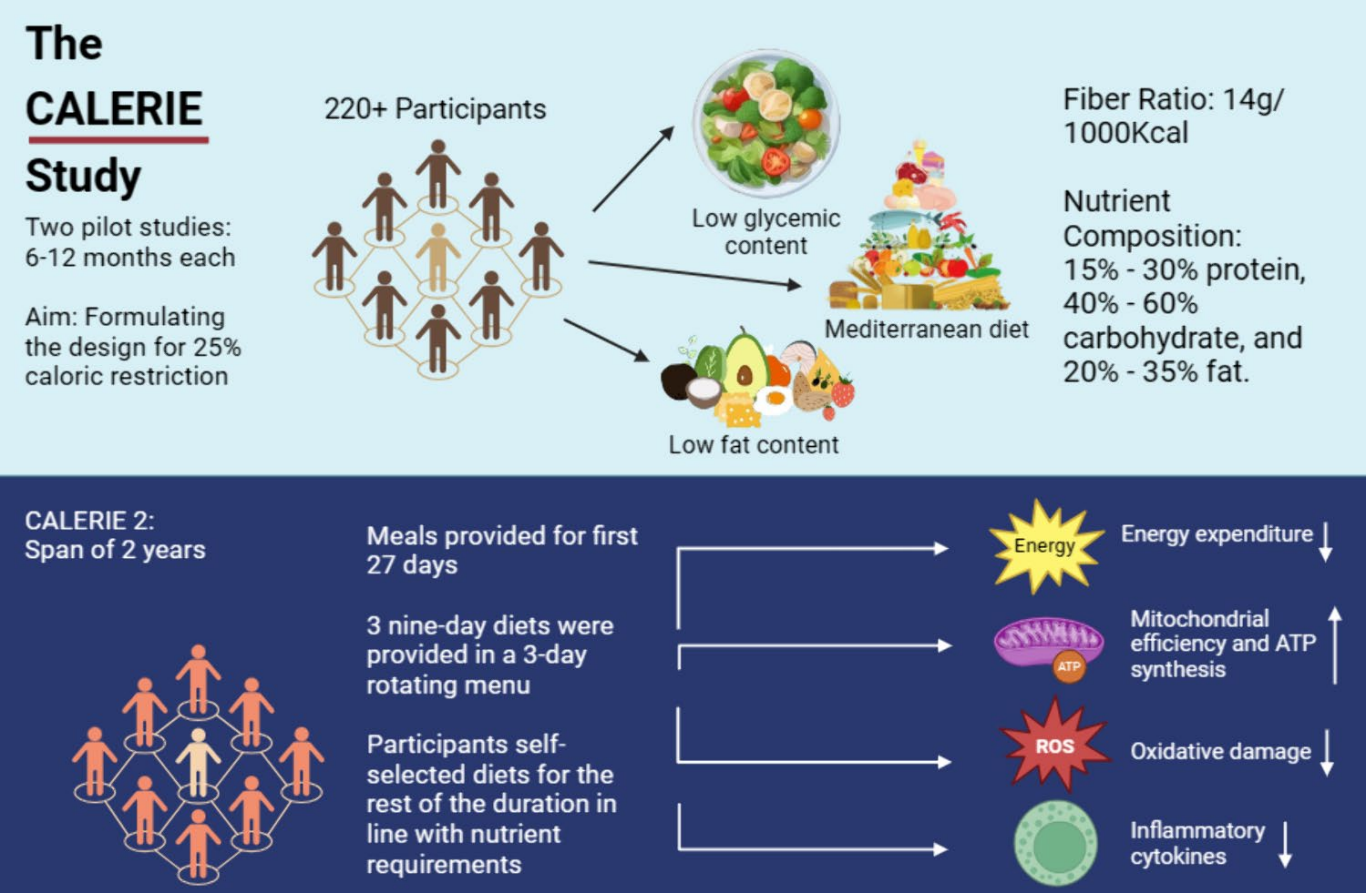
A schematic diagram depicting the methodology and findings of the CALERIE study

The results from the study showed a promising role of CR in managing age-related phenotypes. CR significantly attenuated energy expenditure and increased mitochondrial efficiency (Heilbronn et al. 2006). ATP production was enhanced and the concentration of uncoupling proteins was decreased (Sparks et al. 2017). Moreover, CR reduced oxidative stress as evidenced by examining 2,3-dinor-iPF2αIII, a marker of oxidative stress found in urine (Redman et al. 2018). Additionally, the CR regimen was associated with a marked decline in levels of inflammatory cytokines like interleukins and TNF-α. TNF-α showed a decrease of about 16–20% in only the first two pilot studies. An even more drastic change was observed in all inflammatory markers following the CALERIE 2 trials (Flanagan et al. 2020) (Fig. 3).

Another cohort study sponsored by the University of California, San Francisco assessed the effect of caloric restriction on telomere length. It was a ONE-TIME POINT study having a cross-sectional time perspective. The details of the interventional arms are summarized in Table 1. Telomere length was assessed by drawing blood samples. A 10% decrease was observed in mononuclear blood cells between the CR group and the two controls. However, there is no conclusive indication of the significance of the data. Therefore, follow-up studies with a defined timeframe need to be conducted to further validate whether CR is effective against telomere-induced aging (Long-term Caloric Restriction and Cellular Aging Markers (CRONA) 2017).

**Table 1:** List of all completed and ongoing clinical trials on Caloric Restriction for aging and age-related neurodegenerative disorders (#-Recruiting)

S. No	NCT No	Title of the study	Location	Inclusion criteria	Interventional model and intervention arms	Allocation and masking	Strength	Duration of study	Outcomes	Adverse events
1.	NCT00427193	CALERIE: comprehensive assessment of long-term effects of reducing intake of energy	Duke University Baton Rouge, Louisiana, United States Boston, Massachusetts, United States Saint Louis, Missouri, United States	Age range: Men: 21–50 (Inclusive) Women: 21–47 (Inclusive) 28 > BMI ≥ 22 Females should be using an effective contraceptive throughout the course of the study. These include oral pills, IUDs, or barrier methods No history or clinical manifestation of CVD, Respiratory disorders, Psychiatric disorders, or surgical procedures	Parallel assignment Experimental group: 25% caloric restriction Active Comparator: Control; Ad libitum energy intake	Randomized; single-blind	238	2 years	Change in core body temperature: Not significant Change in resting metabolic rate: Significant; p < 0.0001 Change in TNF-α: Not significant Change in fat mass: Significant; p < 0.001	Serious: 0.7% (Spontaneous miscarriage) Not serious: 95.1% (Anemia, Dizziness, nasal congestion, constipation, toothache, pain, rash, Gastrointestinal virus, UTI, diarrhea, muscle strain, excessive bone loss, dysmenorrhea)
2.	NCT01256840	Long-term caloric restriction and cellular aging Markers (CRONA)	University of California, San Francisco San FranciscoCalifornia, United States	Age > 18 Self-reported caloric restriction for at least two years BMI < 23 No smoking	Cohort observational study	N/A	71	One time point	Telomere T/​S ratio—peripheral blood mononuclear: Not significant	None
3.	NCT05769335#	Calories or time restriction to alter biomarkers of aging and diabetes (OMIT)	University of Adelaide Adelaide, South Australia, Australia	Age: 35–75 years BMI: 25.1–44.9 Elevated fasting glucose > 5.6 mmol/L No history or clinical manifestation of chronic illnesses, gastrointestinal disorders, or psychiatric disorders No medications for diabetes, bet-blockers, glucocorticoids, or anti-epileptics	Parallel assignment Experimental group: 30% early caloric restriction (8:00–16:00) Experimental group: 30% Delayed caloric restriction (12:00–20:00) Active Comparator: Caloric restriction 30% (8:00–20:00)	Randomized; open label	114	2 months	No results; Recruiting Measures include: 24 h glucose onward Insulin AUC Insulin sensitivity Fasting glucose Fasting insulin C-reactive protein Body weight Change in fat mass Physical activity	N/A
4.	NCT05482711#	Assessment of fuel utilization and circadian rhythms in overweight, older adults following time restricted eating—phase 2 (FAR phase 2)	University of Florida Gainesville, Florida, United States	Age > 65 Self-reported difficulty in walking a quarter mile or climbing stairs < 150 min of exercise per week BMI: 25–40 kg/m^2^ No history or clinical manifestations of chronic illnesses No known skin sensitivity or allergies No incidence of angina, heart attack, or stroke in the past 3 months	Single group assignment Time restricted eating intervention: Participants will be asked to stop eating by 7 PM every day and too fast for a target of 16 h per day for 8 weeks	N/A; Open Label	15	8 weeks	No results; Recruiting Measures include: Cellular fuel utilization Daily blood glucose levels Circadian rhythm gene BMAL1 Heart rate Circadian rhythm gene CLOCK Body temperature Circadian rhythm gene Cry1 Circadian rhythm gene per2 Circadian rhythm gene Dbp Circadian rhythm gene Nr1d1 Circadian rhythm gene Nfil2 Physical activity Sleep patterns	N/A
5.	NCT02132091	The effect of intermittent fasting on adaptive oxidative stress response and mitochondrial biogenesis	University of Florida Gainesville, Florida, United States	Age: 19–30 BMI: 20 -30 No history or clinical manifestation of chronic illnesses No alcohol or drug abuse	Crossover assignment Experimental: Intermittent Fasting; Alternate between days of feasting (175% caloric intake) and fasting (25% caloric intake) Experimental group; Intermittent fasting + Antioxidants; Alternate between days of feasting (175% caloric intake) and fasting (25% caloric intake + 400 IU Vitamin E once every morning + 500 mg Vitamin C twice every day	Randomized; quadruple blind	37	3 weeks	No results; completed Measures include: SOD2 gene expression GPx1 gene expression SIRT1 gene expression SIRT3 gene expression mTFA gene expression NRF1 gene expression 8oxodG ratio	N/A
6.	NCT05549362#	Dietary approaches to longevity and health	Pennington biomedical research centre Birmingham, Alabama, United States Baton Rouge, Louisiana, United States	Age: 25–45 BMI ≥ 22 and < 30 Regular sleep–wake cycle Stable eating patterns No history or clinical manifestation of chronic illnesses No psychiatric disorder	Parallel assignment Placebo Comparator: ad libitum energy intake Active Comparator: Traditional CR; 25% in-person sessions Active Comparator: Adaptive CR; 25% using remote, adaptive, and technology-driven intervention Active Comparator: Traditional Time-restricted eating Active Comparator: Adaptive Time-restricted eating	Randomized; Double Blind	90	6 months	No results; recruiting Measures include: CR percent TRE adherence percent	N/A
7.	NCT05732935 #	Fasting to provide energy needed to help adults in need of cognitive enhancement (fasting enhance)	University of Florida Gainesville, Florida, United States	Age ≥ 65 BMI > 25 Evidence of cognitive decline using Subjective Cognitive Decline Questionnaire No history or diagnosis of chronic illnesses No use of anabolic medications No history of drug or alcohol abuse	Parallel assignment Experimental: Time-restricted eating; fasting for 16 h per day Active Comparator: Successful Aging	Randomized; open label	52	24 weeks	No results; recruiting Measures include: Repeatable battery of the assessment of neuropsychological status Montreal cognitive assessment (MoCA) Short physical performance battery (SPPB) Pittsburgh sleep quality index Grip strength Geriatric depression scale Pittsburgh fatiguability scale Glucose regulation (HbA1C), systemic inflammation (C-reactive protein), and Tau State-trait inventory for cognitive and somatic anxiety (STICSA) Grip strength 6 minute walk test	N/A
8.	NCT05698654#	Evaluation of longevity diet and fasting mimicking diet programs on body composition, disease risk factors, and aging markers: a randomized clinical trial	Fondazione Valter Longo Varapodio Calabria, Italy	Age: 30–65 BMI ≥ 25 No history or diagnosis of chronic illness Any of the following: IGF-1 > 200 ng/ml Triglycerides > 150 mg/dl C-reactive protein > 1 mg/L Total cholesterol > 190 mg/dL HbA1C > 5.6%	Parallel assignment Experimental: Fasting mimicking diet; 3 cycles in 6 months Experimental: Longevity diet + Fasting mimicking diet; 3 cycles + longevity diet provided by nutritionist Control: No intervention	Randomized; open label	501	6 months	No results; recruiting Measures include: Change in blood glucose Change in insulin Change in blood glycated hemoglobin Change in LDL, HDL, total cholesterol, and triglycerides Blood pressure Body weight BMI Telomere length Pittsburgh sleep quality index Berlin questionnaire	N/A
9.	NCT02970188	The effect of time-restricted feeding on physiological function in middle-aged and older adults	University of Colorado, Boulder Boulder, Colorado, United States	Age > 55 or ≤ 80 BMI < 40 kg/m^2^ Baseline brachial flow-mediated dilation < 6% Ability to perform motor and cognitive tasks No history or diagnosis of chronic illness No history or clinical manifestation of CVD, thyroid disease, or surgical procedures No alcohol abuse	Crossover assignment Experimental: Time-restricted feeding (8-h window between 10:30/11:30 and 5:30/6:30)	Randomized; open label	12 (estimated)	3 years	No results; unknown status Measures include: Arterial stiffness Cognitive function Motor function Metabolic function	N/A
10.	NCT02460783	Intermittent calorie restriction, insulin resistance, and biomarkers of brain function	National Institute on Aging (NIA) Baltimore, Maryland, United States	Age: 55–70 years BMI ≥ 27 Weight ≤ 350lbs MMSE ≥ 26 HOMA-IR ≥ 1.8 No history or clinical manifestation of other chronic illnesses No history of substance abuse No diabetes No use of systemic corticosteroids No history of psychiatric disorders	Parallel assignment Experimental (CR): caloric restriction: healthy diet for 5 days and CR for 2 days Active comparator (AC): Healthy diet for 7 days	Randomized; open label	129	8 weeks	Change in neuronal derived extracellular vesicle (NDEV)—pS312-IRS-1: not significant Change in NDEV pY-IRS-1: not significant Change in CSF Aβ42: not significant Change in CSF Aβ40: not significant Change in Aβ42/Aβ40 ratio: not significant Change in CSF pTau: not significant Change in CSF Neurogranin: not significant Change in CSF light chain neurofilaments: not significant Change in CSF GFAP: not significant Change in NDEV pAkt/AkT ratio: not significant Change in NDEV α-synuclein: not significant Change in NDEV TDP-43: not significant Change in NDEV MCT1: not significant Change in NDEV mitochondrial complex 4: not significant Change in executive function composite: not significant	Not serious: 40% (Constipation, loose stools, Nausea, Bloating, headache, lightheadedness muscle cramps)
11.	NCT04701957#	The ketogenic diet for Alzheimer’s disease	Assistance Publique—Hôpitaux de Paris, Paris, France	Age ≥ 50 Confirmed AD using CSF sample CDR score = 0.5 Absence of other neurological conditions Absence of chronic inflammation	Parallel assignment Experimental: modified atkins ketogenic diet Control: normal diet	Randomized; single blind	70	12 months	No results; Recruiting Measures include: Cognition using CDR scale Change in weight Change in albumin levels Change in lipid levels Efficiency of brain metabolism Cognition using MMSE Instrumental activities Quality of life using SF-36 scale	N/A

Due to the caveats of CR, newer interventions have been proposed and tested which include time-restricted eating, wherein an individual is limited to consuming foods only at specific times of the day (Mishra et al. 2023). Clinical trials on these alternative diet restriction techniques have been summarized in Table 1 along with their status. These studies aim to introduce interventions that circumvent the toll of complete caloric restriction. Of these studies, one (NCT02460783) assesses the impact of intermittent CR on insulin resistance and biomarkers of brain function. Meanwhile, another study (NCT04701957) focuses on the impact of an alternate dietary structure, the ketogenic diet, to assess its efficiency. Since these trials are still ongoing, there is no indication of its comparative efficiency with traditional CR, but the results are expected to be positive.

## CRMs: Their Classification and Clinical Outlook

**Fig. 4:**
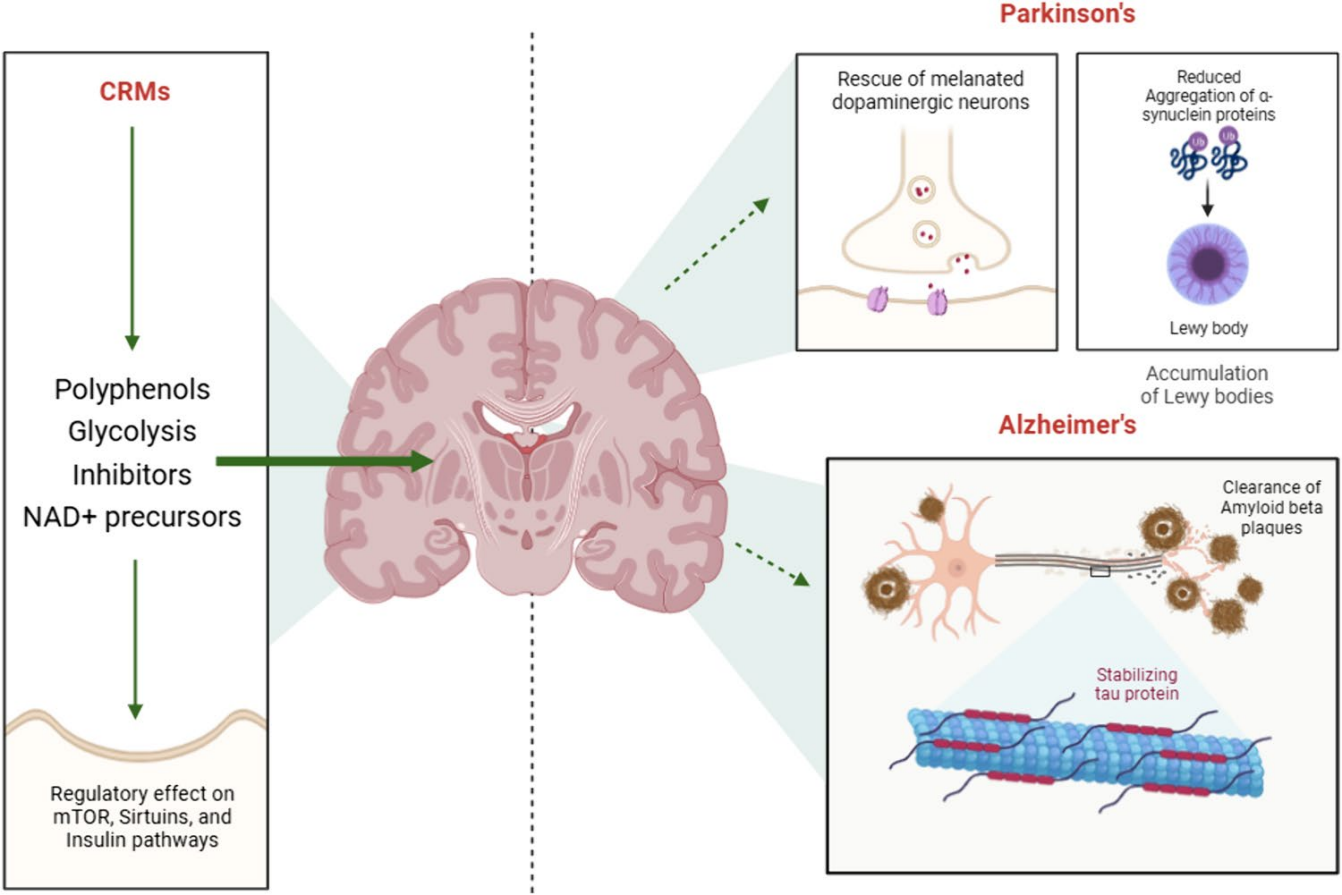
A diagram depicting the alleviation of AD and PD-associated pathophysiological features upon CRM administration

Given the wide range of molecules that have been investigated for their caloric restriction mimetic properties, it is important to elucidate the role of each potential candidate against NDDs. Characterization of the different varieties of CRMs probes the possibility of multi-drug approaches and synergistic effects of these compounds. The major compounds that display properties akin to CRMs are listed in Table 2 and categorically divided into the following groups: Polyphenols, Glycolysis inhibitors, and NAD^+^ precursors (Hofer et al. 2021). The potential therapeutic benefits of CRMs against age-associated neurodegeneration have been summarized in Fig. 4.

**Table 2:** A list of all classes of CRMs and their beneficial effects against neurodegenerative disorders

Group	Caloric restriction mimetic	Mechanism of action	Therapeutic effect on NDDs	References
	Resveratrol	Acts as an anti-inflammatory Reduces ROS generation and has antioxidant properties Decreases levels of IGF-1 Activates Sirt1 by elevation in cAMP signaling Activates AMPK and inhibits mTOR	Reverses cognitive impairment Upregulates neurotransmitter synthesis Promotes neurogenesis Reduces oligomerization of APP Promotes non-amyloidogenic cleavage of APP Upregulates acetylcholinesterase (AChE) activity Alleviates motor defects by increasing dopamine Reduces α-synuclein aggregation	Park et al. (2012), Marchal et al. (2013), Shahidi and Ambigaipalan (2015), Pyo et al. (2020), Zhou et al. (2021), Aslam and Ladilov (2022), Azargoonjahromi and Abutalebian (2024)
Polyphenols	Epigallocatechin gallate	Inhibitor of acetyltransferase Inhibits mitochondrial oxidative phosphorylation Activation of autophagy in a PKA dependent manner Stimulation of AMPK via Ca^2+^/Calmodulin pathway Decreases caloric intake and reduced glucose levels	Inhibits protein aggregation Remodels Aβ oligomers to be non-toxic Slows down α-synuclein fibrillization Reduced α-synuclein mediated toxicity	Wagner et al. (2015), Kim et al. (2017), Madeo et al. (2019), Gonçalves et al. (2021)
	Apigenin	Acts as an anti-inflammatory by reducing expression of COX and NF-κB Increases intracellular NAD + concentration Decreases acetylation of p53 Improves lipid and glucose metabolism Activates ERK pathway Attenuates oxidative stress	Improvement in learning and memory Improvement in sensorimotor coordination Regulation of neurotransmitter levels Reduction in microglial activation Increase in BDNF expression Reduction in caspase-mediated apoptosis Inhibits acetylcholinesterase Inhibits Aβ42 aggregation Prevents loss of dopaminergic neurons Improves mitochondrial functioning	Rezai-Zadeh et al. (2008), Escande et al. (2013), Venigalla et al. (2015), Balez et al. (2016), Liang et al. (2017), Dourado et al. (2020), Siddique et al. (2022), Gaur and Siddique (2024), Olasehinde and Olaokun (2024)
	Quercetin	Suppresses NF-κB inflammation Decreases Histone deacetylases and histone acetyltransferases Reduces DNA methylation Inhibits ROS generation Enhances autophagy Activates SIRT1 Upregulates AMPK signaling	Improves neurogenesis Inhibits α-synuclein fibrillation Improves mitochondrial biogenesis Inhibits β-secretase Inhibits acetylcholinesterase Reduces taupathy Reduces Aβ aggregation Reduces loss of dopaminergic neurons	Costa et al. (2016), Kanter et al. (2016), Amanzadeh et al. (2019), Yessenkyzy et al. (2020), Zu et al. (2021), Deledda et al. (2022), Cui et al. (2022), Chiang et al. (2023), Zhai et al. (2023), Duc Nguyen (2023), Vishwas et al. (2023)
	Curcumin	Inhibits acetyltransferase Autophagy inducer Inhibits COX Reduces NF-κB inflammation Inhibits DNA methyltransferase Affects mTOR, AMPK, PI3K, and SIRT1 pathways Reduces oxidative damage Prevents apoptosis	Ameliorates cognitive decline Reduces microglial activation Promotes neurogenesis and synaptogenesis Inhibits taupathy Inhibits Aβ aggregation Prevents α-synuclein fibrillation Protects dopaminergic neurons	Mariño et al. (2014), Vaiserman et al. (2020), Singh et al. (2023), Nassar et al. (2024)
	Gallic acid	Activates AMPK Activates histone acetyltransferase Induces autophagy Acts as an antioxidant Inhibits caspase-3 mediated apoptosis	Regulates neuro-inflammatory cytokines Balances neuronal signaling by modulating Ca^2+^ signaling Prevents glutamate-mediated excitotoxicity Inhibits amyloid based neurotoxicity Inhibits BACE1 Reduces activity of AChE Reduces loss of dopaminergic neurons	Ekundayo et al. (2022), Bhuia et al. (2023)
	Anthocyanins	Reduces oxidative damage Prevents apoptosis Improves mitochondrial health Maintains Ca^2+^ homeostasis Has anti-inflammatory properties Activates mTOR Enhances autophagy	Improves microglial viability Prevents excitotoxicity Attenuates astrocyte activation Increases BDNF levels Prevents protein aggregation	Winter and Bickford (2019), Li et al. (2021)
	Caffeic acid	Activates Sirt3 Inhibits PI3K/AKT cascade Induces autophagy Improves glucose and lipid metabolism Activates AMPK Alleviates oxidative stress by behaving as an antioxidant Represses immune cytokines like IL-6 and TNF-α Inhibits caspase-3 mediated apoptosis	Reduces neuroinflammation Improves synapse integrity Increases BDNF levels Improves memory Inhibits taupathy	Kulkarni et al. (2021), Alam et al. (2022), Khan et al. (2023)
	Chalcone	Alleviates oxidative stress by behaving as an antioxidant Represses immune cytokines like IL-6 and TNF-α Prevents apoptosis Regulates autophagy	Ameliorates cognitive decline Improves memory Reduces microglial activation Promotes neurogenesis and synaptogenesis Inhibits taupathy Inhibits Aβ aggregation	Chen et al. (2013), Jing et al. (2018), Lee et al. (2018), Xiao et al. (2021), Zhang et al. (2021)
	Berberine	Inhibits PI3/AKT-mTOR cascade Induces autophagy via mTOR control Decreases inflammatory cytokines Activates cellular antioxidant pathways and reduces ROS generation Promotes expression of anti-apoptotic factors	Prevents glutamate-mediated excitotoxicity Causes production of neurotrophic factors Inhibits amyloid-based neurotoxicity by reducing levels of APP and BACE1 Prevents loss of dopaminergic neurons	Jiang et al. (2021), Ahmed et al. (2015), Cai et al. (2016), Chang et al. (2016), Deng and Ma (2021), Cheng et al. (2022), Tian et al. (2023), Fan et al. (2017)
Glycolysis inhibitors	Astragalin	• Activates cellular antioxidant pathways and reduces ROS generation • Promotes expression of anti-apoptotic factors • Reduces levels of inflammatory cytokine like IL-6, IL-1β, and TNF-α Promotes autophagy by regulating Atg genes Reduces mTOR activation	Ameliorates cognitive decline Reduces Aβ aggregation Improves memory by preventing loss of hippocampal neurons Downregulates AChE	Riaz et al. (2018), Yang et al. (2022, 2023), Hu et al. (2022), Chen et al. (2022)
	Metformin	Insulin sensitizer Activates AMPK Causes decline in inflammatory molecules like NF-κB Shows enhanced antioxidant properties by inhibiting mitochondrial complex 1 Inhibits mTOR	Ameliorates the burden of Aβ plaques Improves learning and memory Preserves cognitive function Stabilizes mitochondrial activity Causes Lewy’s body clearance by activation of PP2A (Protein phosphatase 2 A) protein Increases levels of neurotrophic factors like BDNF	Diniz Vilela et al. (2016), Garg et al. (2017), Katila et al. (2017), Pilipenko et al. (2020), Tripathi et al. (2022), Chen et al. (2022)
	Chrysin	Activates cellular antioxidant pathways and reduces ROS generation Reduces levels of inflammatory cytokines like IL-6, IL-1β, and TNF-α Modulates apoptosis Reduces lipid peroxidation and protein carbonylation Alleviates mitochondrial dysfunction	Increases BDNF levels Improves nitric oxide toxicity in Parkinson’s disease Improves serotonin levels Inhibits amyloid based neurotoxicity Downregulates AChE Improves memory by preventing loss of hippocampal neurons Protects dopaminergic neurons	Angelopoulou et al. (2020), Talebi et al. (2021), Mishra et al. (2021), Singh et al. (2024), Goyal et al. (2023)
	Genistein	Inhibits NF-κB mediated inflammatory response Reduces levels of circulating interleukins Reduces oxidative stress by activating intracellular antioxidants Reduces apoptotic markers like Caspase 9	Inhibits amyloid-based neurotoxicity by reducing levels of APP and BACE1 Improves blood brain barrier function Reduces astrocyte activation Inhibits tau hyperphosphorylation Improves memory and cognitive functioning	Ye et al. (2017), Uddin and Kabir (2019), Duan et al. (2021), Mas-Bargues et al. (2022), Li et al. (2022), Gao et al. (2022), Paramanik et al. (2023)
	Mannoheptulose	Inhibits glucokinase, reducing glycolysis Activates AMPK Increases NAD+ levels, activating Sirt1	Reduces oxidative stress Improves mitochondrial function Decreases amyloid-β levels	Ingram and Roth (2021)
	Acarbose	Inhibits intestinal α-glucosidases, slowing carbohydrate absorption Reduces hyperglycemia	Decreases inflammation Improves insulin sensitivity Reduces risk of dementia	Tong et al. (2015), Sonsalla et al. (2024)
	D-glucosamine	Inhibits the hexosamine biosynthetic pathway Reduces O-GlcNAcylation of proteins Activates AMPK	Reduces tau hyperphosphorylation Improves synaptic function Decreases neuroinflammation	Castellani et al. (2007)
NAD + Precursors	Nicotinamide	Reduces oxidative damage Prevents apoptosis by reducing levels of caspase-3, caspase-9, and cytochrome C Prevents mitochondrial damage Improves Insulin resistance Mediates autophagy by reducing mTOR Activates AMPK pathways Activates SIRT1	Prevents glutamate-mediated excitotoxicity Causes production of neurotrophic factors Reduces Aβ toxicity by facilitating clearance of senile plaques Reduces microglial activation Prevents loss of dopaminergic neurons	Fricker et al. (2018), Salech et al. (2020), Maiese (2021), Vreones et al. (2023), Zhao et al. (2023)
	Niacin	Reduces oxidative damage Reduces inflammatory cytokines like IL-6, IL-1β, and NF-κB Reduces oxidative stress by mediating the role of mitochondrial complexes Regulates mTOR signaling	Contributes to increasing dopamine production Promotes positive immune response via activation of macrophages and reduction in microglial activation Reduces amyloid formation	Giri et al. (2019), Gasperi et al. (2019), Wuerch et al. (2023)
	Oxaloacetic acid	Inhibits malate dehydrogenase, altering TCA cycle Increases NAD+ levels Activates Sirt1	Enhances neurogenesis Improves cognitive function Reduces neurodegeneration	Li et al. (2023a, b)

To assess the efficacy of Rapamycin in humans, several clinical trials have been conducted, both targeting aging and neurodegenerative disorders. The clinical trial titled “Effect of mTOR Inhibition and Other Metabolism Modulating Interventions on the Elderly” (NCT02874924) measured the effect of Rapamycin on immunological functioning and its cardiovascular consequences. The study consisted of two arms: a Rapamycin group (1 mg Rapamycin daily), a Placebo group, and an additional sub-study where 1 mg Rapamycin was administered every day and cardiovascular parameters including diastolic function were measured. The duration of the trial was 8 weeks. While the trial did report an increase in T-cell function, statistical analysis revealed that the difference was not significant. Moreover, no difference was observed in walking speed signifying a lack of improvement in physical performance. EXIT25 (The executive Interview) is a test used to assess executive functioning scored between 0 to 50 with a score below 15 indicating normal executive functioning. EXIT25 also revealed that there was no difference between the Rapamycin group and the placebo group. The lack of positive results, in the case of this specific trial, can be attributed to the low dose (1 mg) of Rapamycin as the ideal dosage at which Rapamycin shows protective effects without adverse side effects is listed to be between 5 and 7 mg. It has been established that a 1 mg dosage is suboptimal in producing the desired effects (Blagosklonny 2023). Moreover, the duration of the trial limits its precision as the protective effects of rapamycin may take longer to manifest. Lastly, a smaller sample size amongst which not all completed the regime could potentially contribute to the lack of positive results.

Many clinical trials targeting both aging and neurodegenerative disorders have been carried out to evaluate the effectiveness of Metformin in humans. The MILES study (NCT02432287) is a Phase 4 study that was conducted to analyse transcriptomic changes by employing RNA sequencing of genes expressed in muscles and adipose tissue. The study also monitored insulin sensitivity and insulin secretion using a modification of the Matsuda Index with a higher index indicating high sensitivity. The details of interventional arms are mentioned in Table 3. Group 1 showed an upregulation in 245 genes in the muscle and 15 genes in adipose tissues whereas group 2 exhibited upregulation in 402 and 132 genes in muscles and adipose tissue respectively. While there is an increase in several numbers of genes modulated, the results are not statistically significant, necessitating the need for a broad-range study with more participants and longer durations.

**Table 3:** List of all clinical trials on the CRMs against age-related neurodegenerative disorders (#-Recruiting)

S. No	NCT No	Title of the study	Sponsor and location	Inclusion criteria	Interventional Model Intervention arms	Allocation and masking	Strength	Duration of study	Outcomes	Adverse events
		Metformin								
1.	NCT02432287	Metformin in longevity study (MILES)	Albert Einstein College of Medicine Bronx, New York, United States	Age: ≥ 60 years Impaired glucose tolerance and Impaired fasting glucose No serious chronic illnesses No substance abuse	Crossover assignment Experimental: 1700 mg metformin daily Experimental: Placebo	Randomized; double blind	16	6 weeks	Increase in gene expression in Muscle: No measure of significance and adipose tissue: No measure of significance Insulin sensitivity and Insulin secretion: No measure of significance	Serious: 6.67% (Nausea, urinary retention, hematoma)
2.	NCT03309007	A double-blind, placebo-controlled trial of anti-aging, pro-autophagy effects of metformin in adults with prediabetes—phase 3	University of New Mexico Albuquerque, New Mexico, United States	Age: 30–70 years Prediabetes as measured by HbA1c levels between 5.7 and 6.4% BMI: 27–40 kg/m^2^	Parallel assignment Experimental: 500 mg Metformin twice daily initially. Titrated up to 1000 mg in the morning and 500 mg in the evening as per the subject’s tolerance Placebo comparator: CaCO_3_ as placebo	Randomized; quadruple blind	25	12 weeks	Change in leucocyte LC3: No measure of significance	Not serious: 7.69% (Diarrhea)
3.	NCT01765946	Metformin and longevity genes in prediabetes—phase 4	University of Padova Padova, Italy	Age: 40–75 years Impaired glucose tolerance and Impaired fasting glucose Absence of chronic illnesses Absence of immunological diseases	Parallel assignment Experimental: 500 mg Metformin Placebo Comparator: Placebo tablets	Randomized; single blind	38	2 months	No results; completed Measures include: Longevity genes (Sirtuin-1, p66Shc, mTor, p53) expression Insulin sensitivity Monocyte polarization	N/A
4.	NCT04264897#	Does insulin sensitivity impact the potential of metformin to slow aging	Oklahoma medical research foundation Oklahoma City, Oklahoma, United States Madison, Wisconsin, United States	Age: 40–75 years No chronic diseases No CVD No Diabetes No neurological diseases No substance abuse	Parallel assignment Experimental: Metformin Week 1–500 mg Week 2–1000 mg Week 3–1500 mg Placebo comparator: Placebo oral pills	Randomized; triple blind	148	12 weeks	No results; Recruiting Measures include: Insulin sensitivity Change in mitochondrial function of the ETS Change in glucose levels Change in blood-based biomarkers for aging (HbA1c, glucose, and insulin)	N/A
5.	NCT02745886	Chronic metformin treatment induces a dietary restriction-like state in overweight human—phase 4	Xiang Guang-da, Wuhan General Hospital of Guangzhou Military Command Wuhan, Hubei, China	Age: 18–60 years Male candidates No obesity No mental disorders No substance abuse	Parallel assignment Experimental: metformin 0.85 twice a day No intervention: standard diet CR group: caloric restriction	Randomized; open label	60	6 months	No results; unknown status Measures include: Difference in gene expression profile Insulin sensitivity	N/A
6.	NCT01965756	A phase II trial to study the effect of metformin on AD biomarkers: a randomized placebo controlled crossover pilot study of metformin effects on cognitive, physiological and biochemical biomarkers of MCI and dementia due to AD	University of Pennsylvania Philadelphia, Pennsylvania, United States	Age: 55–80 years Diagnosis of MCI or early dementia No history of diabetes No mental disorders No psychiatric illnesses No major lesions	Crossover assignment Experimental: Metformin (8 weeks; starting with 500 mg and increasing 500 mg per week till 2000 mg is reached), then placebo (8 weeks) Experimental: Placebo (8 weeks), then metformin (8 weeks; starting with 500 mg and increasing 500 mg per week till 2000 mg is reached)	Randomized; quadruple blind	20	16 weeks	CSF Aβ concentration: not significant Total Tau concentration: not significant CSF pTAU concentration: not significant	Not serious: 10% (Elevates plasma lactate)
7.	NCT00620191	Metformin in the prevention of Alzheimer’s disease—phase 2	Columbia University New York, New York, United States	Age: 55–90 years Self-reported memory impairment MMSE ≥ 20 Diagnosis of MCI Global Clinical Dementia Rating = 0.5 Absence of other neurological diseases No psychiatric illness No known history of diabetes medication	Parallel assignment Placebo comparator: 2 tablets Experimental: 1000 mg metformin twice a day	Randomized; quadruple blind	80	12 months	Change in Total Recall Score: Not significant Change in ADAS-cog score: Not significant Change in glucose uptake in posterior cingulate precuneus: Not significant Change in plasma Aβ42: Not significant	None
8.	NCT04098666#	Metformin in Alzheimer’s disease prevention	Columbia University Irvine, California, United States La Jolla, California, United States Palo Alto, California, United States Washington, District of Columbia, United States	Age: 55–90 years Diagnosis of MCI MMSE ≥ 22 CDR = 0.5 BMI > 20 kg/m^2^ No history of metformin tolerance No diagnosis of dementia No neurologic diseases No CVD No substance abuse No history of cancer	Parallel assignment Experimental: Metformin extended release 500 mg tablets, up to 4 tablets a day Placebo Comparator: Upto 4 tablets a day	Randomized; quadruple blind	326	18 months	No results; recruiting Measures include: Free and cued selective reminding test (FCSRT) Alzheimer’s disease cooperative study preclinical Alzheimer’s cognitive composite Cortical thickness White matter hyperintensity volume Brain Aβ Brain Tau Plasma Aβ Plasma Tau Plasma light neurofilament	N/A
9.	NCT05781711#	Clinical study to evaluate the possible efficacy of metformin in patients with Parkinson’s disease—phase 2	Tanta University Tanta, Shebeen El-Kom, Egypt	Age ≥ 18 years Diagnosis of PD Taking Levodopa No anti-inflammatory drugs No substance abuse No known allergy to medications	Parallel assignment Experimental: Control group levodopa/carbidopa (50/250 mg) thrice daily Active Comparator: levodopa/carbidopa (50/250 mg) thrice a day + 500 mg Metformin twice a day	Randomized; double blind	60	3 months	No results; recruiting Measures include: Change in the unified Parkinson’s disease rating scale (UPDRS) Changes in serum biomarkers (BDNF)	N/A
		Rapamycin								
7.	NCT02874924	Effect of mammalian target of rapamycin inhibition and other metabolism modulating interventions on the elderly: immune, cognitive, and functional consequences—phase 2	The University of Texas Health Science Center at San Antonio San Antonio, Texas, United States	Age: 70–95 years All chronic illnesses are stable Adequate cognitive functioning as assessed by CLOX1 score (≥ 10) No history or clinical manifestation of CVD No current diagnosis of hypercholesteremia, hypertriglyceridemia, or diabetes No substance abuse	Parallel assignment Experimental: 1 mg Rapamycin (Sirolimus) Placebo comparator: control	Randomized; quadruple blind	34	8 weeks	Change in Immunological Response: not significant Change in cognitive function: not significant	Not serious: 14.29% (Diarrhea, Face rash)
8.	NCT04488601	Participatory evaluation (of) aging (with) rapamycin (for) longevity study (PEARL): a prospective, double-blind, placebo-controlled trial for rapamycin in healthy individuals assessing safety and efficacy in reducing aging effects	AgelessRx Chicago, Illinois, United States	Age: 50–85 years Stable chronic diseases Absence of anemia Absence immunotherapy Absence of psychiatric disorders Absence of CVD	Parallel assignment Experimental: 5 mg Rapamycin/week Experimental: 10 mg Rapamycin/week Placebo comparator: control	Randomized; quadruple blind	129	12 months	No results; completed Measures include: Change in visceral fat Bone density Lean mass Complete blood count Change in blood electrolytes Change in serum bilirubin Change in serum alkaline phosphatase Change in serum albumin Change in cholesterol Change in insulin Change in IGF-1 Change in HbA1c	N/A
9.	NCT04742777#	Effect of mTOR inhibition and other metabolism modulating interventions on the elderly: immune, cognitive, and functional consequences ((Substudy E-RAPA cMRI with LGE)	The University of Texas Health Science Center at San Antonio San Antonio, Texas, United States	Age: 70–95 years Stable chronic diseases Absence of anemia Absence immunotherapy Absence of psychiatric disorders Absence of CVD COVID negative	Single group assignment Experimental: 1 mg rapamycin	N/A; open label	12	8 weeks	No results; recruiting Measures include: Systolic function Diastolic function Aortic cross-sectional area Aortic distensibility	N/A
10.	NCT04200911	Cognition, age, and rapamycin effectiveness—downregulation of the mTOR pathway (CARPE DIEM)	The University of Texas Health Science Center at San Antonio San Antonio, Texas, United States	Age: 55–85 years Diagnosis of mild cognitive impairment No diabetes No other significant neurological deficit No chemotherapy	Single group assignment Experimental: 1 mg Rapamune	N/A; open label	10	8 weeks	Change in CSF Aβ42: not significant Change in clinical dementia rating:: not significant Change in neuropsychiatric inventory:: not significant	Serious: 10% (altered mental status) Not serious: 40% (cough, upper respiratory infection, Diarrhea, UTI)
11.	NCT04629495#	Rapamycin—effects on Alzheimer’s disease and cognitive health (REACH)—phase 2	The University of Texas Health Science Center at San Antonio San Antonio, Texas, United States	Age: 55–85 years Diagnosis of Mild cognitive impairment or AD Amyloid positivity as assessed by PET No other chronic illnesses No CVD No chemotherapy or radiotherapy	Parallel assignment Active comparator: 1 mg RAPA daily Placebo comparator: Placebo capsule	Randomized; quadruple blind	40	12 months	No results; recruiting Measures include: Change in glucose levels Change in serum albumin Change in bicarbonate levels Change in calcium levels CNS penetration of RAPA Change in CDR rating Change in Cognition using preclinical Alzheimer’s Cognitive Composite 5 Change in Neuropsychiatric symptoms Change in brain volumetry Change in cerebral glucose metabolism Change in CSF Aβ levels	N/A
12.	NCT01649960	Exercise and low-dose rapamycin in older adults with CAD: cardiac rehabilitation and rapamycin in elderly trial (CARE)	Mayo clinic Rochester, Minnesota, United States	Age: > 60 years Able to undergo cardiac rehabilitation No class III or IV CHF Creatinine > 2.0 mg/dl HbA1c > 13% Absence of anemia Absence of stroke Absence of psychiatric disorders Absence of CVD COVID negative	Single group assignment Participant group: Oral tablets of rapamycin were given in the dose of 0.5 mg, 1 mg, or 2 mg once a day	Open label; N/A	13	12 months	No results; completed Measures include: Frailty Senescence associates secretory phenotypes like interleukin-6, matrix metalloproteinase 3, and monocyte chemotactic protein 1 Quality of life using short-form 12 Mitochondrial DNA number	N/A
		Resveratrol								
13.	NCT02523274	Resveratrol and exercise to treat functional limitations in late life	University of Alabama, Birmingham, United States	Age: ≥ 65 years Physical limitations Sedentary lifestyle No CVD No neuropathy Pain < Grade 3 No instance of dementia	Parallel assignment Placebo Comparator: Placebo + Exercise Experimental (RV500): 500 mg Resveratrol/day + Exercise Experimental (RV1000): 1000 mg Resveratrol/ day + Exercise	Randomized; double blind	60	12 weeks	Physical Performance Battery: No measure of significance Isokinetic strength: No measure of significance 6 min walk test: No measure of significance Late life function and Disability instrument: No measure of significance	Not serious: 40% (GI issues, light-headedness, bladder infection, musculoskeletal disorder, blood sugar, bronchitis, skin rash, elevated BP)
14.	NCT05500742	The proBNPage reduction (PBAR) randomized trial: a pilot study to define the characteristics of future randomized trials aimed at evaluating the effects of anti-aging treatments on a surrogate of biological age in healthy older adults	IRCCS Azienda Ospedaliero-Universitaria di Bologna Bologna, Italy	Age: 65–80 years No history of CVD No prescription of anticoagulants No known allergy No chronic illnesses No substance abuse	Parallel assignment Active comparator: 100 mg coenzyme Q10 + 100mcg Selenium Active comparator: 350 mg resveratrol + 100U TA-65 Placebo comparator: placebo	Randomized; double blind	120	24 months	No result; active, not recruiting Measures include: Change in ProBNPage	N/A
15.	NCT05981053	Biological effects in consumers of resveratrol-enriched wine	PINER Pastor Manfredi Winery Buenos Aires, Buenos Aires City, Argentina	Age: 40–80 years Regular wine consumers No pregnancy No alternative treatments No chronic diseases No psychiatric illness No epilepsy	Case-only prospective observational study	N/A	30	3 months	No results; completed Measures include: Epigenetic age Muscle mass Body composition	N/A
16.	NCT00823381	Effect of resvida (tm) dietary supplementation on muscle gene expression: a comparison with calorie restriction regimen	Washington University School of Medicine St. Louis, Missouri, United States	Age: 35–70 years Post-menopausal females 30 > BMI ≥ 20 No CVD No allergy Sedentary lifestyle No substance abuse No chronic illness	Parallel assignment Experimental: 75 mg resveratrol Placebo comparator: Placebo Active comparator: 30% caloric restriction	Randomized; triple blind	58	3 months	No results; completed Measures include: Global skeletal muscle gene expression profile Insulin Sensitivity Blood lipid levels Inflammatory markers Intrahepatic triglyceride content	N/A
17.	NCT02123121	Phase IIa study of resveratrol to enhance mitochondrial and physical function in older adults	University of Florida Gainesville, Florida, United States	Age ≥ 65 years Moderate to high functioning BMI: 20–39.9 kg/m^2^ No allergy to grapes or knotweed No consumption of dietary supplements No CVD No cognitive impairment No substance abuse Fasting glucose < 126 mg/dL	Parallel assignment Placebo comparator: vegetable cellulose Active comparator: resveratrol 1000 mg/day Active comparator: resveratrol 1500 mg/day	Randomized; double blind	60	9 months	No results; completed Measures include: Mitochondrial respiration in muscles Change in muscle COX Change in muscle citrate synthase Change in mtDNA content Change in PGC-1α muscle protein Blood glucose Change in muscle AMPK Change in muscle Sirt1 and Sirt3	N/A
18.	NCT01504854	Phase II study to evaluate the impact on biomarkers of resveratrol treatment in patients with mild to moderate Alzheimer’s disease	Alzheimer’s disease cooperative study (ADCS) Phoenix, Arizona, United States Irvine, California, United States La Jolla, California, United States Los Angeles, California, United States	Age ≥ 50 years Diagnosis of AD MMSE: 14–26 Modified Hachinski score ≤ 4 No chronic illnesses No neoplastic disease No history of stroke or seizure	Parallel assignment Experimental: resveratrol 500 mg Placebo comparator: placebo	Randomized; quadruple blind	119	52 weeks	Volumetric MRI: No measure of significance Activities of daily living for mild cognitive impairment scale: no measure of significance CSF Aβ40: no measure of significance	Serious adverse events: 20.31% (syncope, Perforated viscus, UTI, hypokalemia, Rhabdomyolosis, Bladder tumor, Seizure, altered mental status, Pulmonary embolism, Right peripheral vascular disease) Not serious: 100% (Diaarhea, UTI, Dizziness, Headache, Anxiety, Raah, Hypertension)
19.	NCT00678431	A single center, multi-site, randomized, double-blind, placebo-controlled trial of resveratrol with glucose and malate (RGM) to slow the progression of Alzheimer’s disease	US Department of Veterans Affairs Bronx, New York, United States	Age: 55–90 years Probable AD MMSE: 12–26 Modified Hachinski < 4 No CVD No chronic illnesses No diabetes No psychiatric disorder No neoplastic disease	Parallel assignment Placebo comparator: liquid placebo Experimental: liquid resveratrol with glucose, and malate	Randomized; quadruple blind	27	1 year	No results; completed Alzheimer disease assessment scale (ADAScog) CGIC	N/A
		Quercetin								
21.	NCT05422885#	Senolytics to alleviate mobility issues and neurological impairment in aging	Lewis Lipsitz, Hebrew SeniorLife Boston, Massachusetts, United States	Age ≥ 62 years Mild cognitive Impairment Ambulatory No allergies No chronic illness No other neurological disorder	Single group assignment Experimental: 100 mg Dasatinib for 2 days every 2 weeks 1250 mg Quercetin for 2 days every two weeks	N/A; open label	12	14 weeks	No results; recruiting Measures include: Neurovascular coupling Executive functions Gait speed Physical performance Mobility and grip strength Senescent cells (p16 expression) SASP in blood and urine (IL-1α, IL-6, MMP-9, and MMp-12)	N/A
22.	NCT05838560#	Dasatinib plus quercetin for accelerated aging in mental disorders	Washington University School of Medicine Saint Louis, Missouri, United States	Age ≥ 50 years Atleast three conditions associated with aging No history of dementia Taking an adequate dose of medication for schizophrenia/schizoaffective disorder or depression Not taking medications that are strong CPY3A4 inhibitors or strong inducers, or that induce senescence No other chronic illness	Single group assignment Experimental: 100 mg Dasatinib + 1250 mg Quercetin	N/A; open label	40	10 weeks	No results; recruiting Measures include: Safety and feasibility of dasatinib plus quercetin	N/A
23.	NCT04063124	Pilot study to investigate the safety and feasibility of senolytic therapy to modulate progression of Alzheimer’s Disease (SToMP-AD)	The University of Texas Health Science Center at San Antonio San Antonio, Texas, United States	Age: ≥ 65 years Clinical diagnosis of AD BMI: 19–35 kg/m^2^ Normal labs No alcohol or drug abuse No chronic illnesses No anti-arrhythmic medications No current use of quinolones No lesions on MRI	Single group assignment Experimental: Dasatinib + Quercetin 2 days on/14 days off for 12 weeks	N/A; open label	5	12 weeks	Results posted Measures include: Brain penetration of Dasatinib and Quercetin CSF tau CSF Aβ Senescence marker (IL-6, P16) Gait mapping Montral cognitive assessment	N/A
24.	NCT04685590#	Phase II clinical trial to evaluate the safety and feasibility of senolytic therapy in Alzheimer’s disease	Wake Forest University Health Sciences Winston-Salem, North Carolina, United States Barcelona, Spain Sevilla, Spain	Age ≥ 65 years Diagnosis of MCI or early AD Elevated tau Normal labs BMI < 40 Normal MRI No chronic illnesses No neurological diseases other than AD Not taking medications that induce senescence	Parallel assignment Experimental: 100 mg Dasatinib for 2 days + 4 × 250 mg Quercetin for 2 days Placebo comparator: Placebo capsules	Randomized; quadruple blind	48	48 weeks	No results; recruiting Measures include: Change in SASP biomarkers (cyclin-dependent kinase inhibitor 2A, CD3, T cells) Change in clinical dementia rating scale Change in Alzheimer's disease assessment scale—cognitive subscale Change in tau pathology detected by PET	N/A
		Curcumin								
25.	NCT03085680	Does dietary supplementation with curcumin maintain or improve physical and cognitive function in aging adults at increased risk for disability?	University of Florida Gainesville, Florida, United States	Age: 65–99 years Usual walking speed < 1 m/sec and > 0.44 m/sec CRP > 1.0 mg/dl MMSE > 24 BP < 160/100 No chronic illnesses No neurological conditions No alcohol or substance abuse No diabetes No use of anticoagulants No use of anabolic medications	Parallel assignment Experimental: 1000 mg Curcumin per day: 2 capsules before breakfast Placebo Comparator: Microcrystalline cellulose	Randomized; double blind	17	3 months	Walking speed: Not significant Hand grip: Not significant Attention and memory: not significant Pain: not significant Interleukin-6: Not significant	None
26.	NCT00099710	A phase II, double-blind, placebo-controlled study of the safety and tolerability of two doses of curcumin c3 complex versus placebo in patients with mild to moderate Alzheimer’s disease	John Douglas French Foundation Westwood, California, United States	Age ≥ 50 years Diagnosis of probable AD Sporadic AD; Not familial No neurological or psychiatric disorders No chronic illnesses No alcohol or substance abuse	Parallel assignment Dietary supplement: curcumin C3 complex	Randomized; double blind	33	12 months	No results; completed Measures include: Oxidative damage Inflammation/gliosis Aβ levels Tau levels LDL, HDL, and total cholesterol Plasma curcumin and metabolites Cognitive and behavioral measures	N/A
27.	NCT00164749	A pilot study of curcumin and ginkgo for treating Alzheimer’s disease	Chinese University of Hong Kong Shatin, Hong Kong	Age ≥ 50 years Decline in memory and cognitive function for at least 6 months No drug abuse or active smokers No other chronic illness	Parallel assignment Placebo comparator: color-matched placebo + 120 mg/day ginkgo extract Experimental: 1 gm curcumin/day + 120 mg/day ginkgo extract Experimental: 4 gm curcumin/day + 120 mg/day ginkgo extract	Randomized; double blind	36	6 months	No results; completed Measures include: Change in MMSE score Change in plasma isoprostane levels Change in serum Aβ Change in cholesterol and triglycerides Change in serum metals (after 1 month) Level of curcumin in plasma vs. dose (after 1 month)	N/A
		Anthocyanins								
28.	NCT04348162	Food anthocyanins and flavanols as a strategy for a healthy ageing: cardiovascular health and cognitive performance	National Research Council, Spain Madrid, Spain	Age: 50–85 years BMI: 20–32 kg/m^2^ MMSE > 28 Systolic BP < 139 mmHG Diastolic BP < 89 Total cholesterol < 6.5 mmol/L	Parallel assignment Active comparator: cocoa flavanols Active comparator: berries anthocyanins Active comparator: cocoa flavanols + berries anthocyanins No intervention: control	Randomized; double blind	90	10 weeks	No results; completed Measures include: Flow-mediated vasodilatation (FMD) of the brachial artery STROOP score Tower of London score	N/A
29.	NCT03824041	Aronia berry supplementation for improving vascular endothelial dysfunction and modulating the gut microbiota in middle-aged/older adults	Colorado State University Fort Collins, Colorado, United States	Age: 45–75 years Hemoglobin A1C ≤ 6.4% Blood pressure < 129/80 mmHg Total cholesterol < 240 mg/dL LDL Cholesterol < 190 mg/dL Triglycerides < 350 mg/dL BMI ≥ 18.5 and < 30 kg/m^2^ No chronic illnesses No use of antibiotics No alcohol abuse No active smokers	Crossover assignment Placebo Comparator: Placebo Experimental: 500 mg 50% Aronia full spectrum + 50% placebo twice a day Experimental: 500 mg 100% Aronia full spectrum twice a day	Randomized; double blind	24	6 weeks	No results; completed Measures include: Reactive hyperemia index (RHI) Gut microbiota analysis Blood pressure Augmentation index Gastrointestinal health Pulse Wave Velocity Blood lipids Blood Hemoglobin A1C Blood oxidized LDL	N/A
30.	NCT04084457	Investigating the effects of daily consumption of blueberry (poly)phenols on vascular function and cognitive performance (BluFlow)	King’s College London London, United Kingdom	Age 65–80 years No cardiovascular diseases No diabetes BMI < 30 No active smokers No history of chronic illnesses No psychological conditions	Parallel assignment Placebo comparator: Placebo Active comparator: 27 g wild blueberry powder	Randomized; double blind	53	12 weeks	No results; completed Measures include: Endothelial function Cognitive function Cerebral blood flow velocity Office systolic pressure Pulse wave velocity Augmentation index Mood Change in lipids Microbiome analysis Pulsatility index Plasma blueberry polyphenol metabolites	N/A
		Genistein								
31.	NCT01982578	Effect of activation of the receptor PPARg/RXR as a possible treatment for Alzheimer’s disease. Role of genistein	Fundación para la Investigación del Hospital Clínico de Valencia Valencia, Spain	Age > 18 years MCI compatible with prodromal AD MMSE > 24 CSF levels of Aβ and Tau compatible with AD No thyroid abnormalities No immune abnormalities	Parallel assignment Experimental: 60 mg genistein BID for 1 year Placebo Comparator: 1 Placebo capsule for 1 year	Randomized; quadruple blind	27	1 year	No results; completed Measures include: Change in CSF Aβ Change in MMSE Change in T@M (Memory alteration test) Changes in clock test Changes in TAVEC Change in Barcelona test Change in Rey complex figure test	N/A
Nicotinamide										
32.	NCT00580931	Efficacy of Nicotinamide for the Treatment of Alzheimer’s Disease	University of California, Irvine Irvine, California, United States	Age: 50 – 95 years MMSE: 13–25 Brain imaging consistent with probable AD Hachinski Ischemic score < 4 No dementia No other neurological illnesses No psychiatric illnesses No alcoholism	Parallel Assignment Experimental: 1500 mg nicotinamide twice a day Placebo Comparator: Enduramide placebo twice a day	Randomized; quadruple blind	50	6 months	No results; results submitted Measures include: Alzheimer’s disease assessment scale-cognitive subscale	N/A
33.	NCT03061474	A double-blind-randomized, placebo-controlled adaptive design trial of nicotinamide in mild cognitive impairment due to Alzheimer’s disease and mild Alzheimer’s disease dementia	University of California, Irvine Irvine, California, United States Los Angeles, California, United States	Age ≥ 50 years MCI or AD-dementia CSF Aβ ≤ 600 pg/ml Tau to Aβ ratio ≥ 0.39 MMSE ≥ 20 Hachinski Scale < 4 Geriatric Depression Scale < 6 No chronic illnesses MRI evidence of stroke < 1 cm	Parallel assignment Experimental: 750 mg Nicotinamide twice a day Placebo Comparator: 750 mg Placebo twice a day	Randomized; double blind	46	48 weeks	Change in pTau: not significant Change in Aβ40: not significant Change in Aβ42: not significant Change in pTau181: not significant Change in total Tau: not significant Change in ratio of total Tau/ Aβ40: not significant Change in total Tau/Aβ42: not significant ADASCog13: not significant Mild cognitive impairment: not significant CDR sum of boxes: not significant	Serious: 8.33% (Increased transaminase, Colorectal cancer) Not serious: 95.83% (Colitis ulcerative, diarrhea, constipation, dyspepsia, eructation, hiatus hernia, vomiting, fungal infection, pneumonia, UTI, viral upper respiratory tract infection, Decreased blood testosterone, abnormal lab tests, decreased GFR, dehydration, diabetes mellitus, Vit. D deficiency, Myalgia, Osteoporosis, basal cell carcinoma, anxiety, depression, Hematuria, urinary retention, dizziness, tremors, psoriasis, rash, dysgeusia)
34.	NCT04430517#	Effects of orally administered nicotinamide riboside on bioenergetic metabolism, oxidative stress and cognition in mild cognitive impairment and mild Alzheimer’s dementia	Mclean Hospital Belmont, Massachusetts, United States	Age: 55–89 years Diagnosis of MCI or AD One copy of the APOE ε4 allele or AD + including Amyloid positive PET scan, Tau positive PET Scan, or CSF AD biomarkers No alcohol or substance abuse No chronic illnesses No use of parkinsonian medication No history of seizures No use of medications with known adverse cognitive effects No use of antioxidants or mitochondrial enhancers	Single group assignment 250 mg Nicotinamide Riboside 4 pills per day	N/A; open label	50	12 weeks	No results; recruiting Measures include: Brain NAD + Brain redox state Mitochondrial functioning Brain GSH Cognitive status Functional status Mood Behavioral or psychiatric symptoms Spirituality or religious beliefs	N/A
35.	NCT05617508#	N-DOSE AD: A dose optimization trial of Nicotinamide Riboside (NR) in Alzheimer’s disease	Haukeland University Hospital Bergen, Vestland, Norway	Age: 50–85 years Diagnosis of probable AD according to NIA criteria Biomarkers of AD in CSF Clinical Dementia Rating: 0.5–1 MoCA ≥ 16 or MMSE ≥ 20 Able to undergo MRI No other comorbidities No anticoagulants No psychiatric disorder	Parallel assignment Placebo Comparator: Placebo Experimental: 500 mg NR capsule twice a day Experimental: Dose escalation group—1000 mg NR daily in doses of 500 mg twice daily (week 1–4); 2000 mg NR daily in doses of 1000 mg twice daily (week 5–8); 3000 mg NR daily in doses of 1500 mg twice daily (week 9–12)	Randomized; triple blind	80	12 weeks	No results; recruiting Measures include: Cerebral nicotinamide adenine dinucleotide (NAD) levels CSF NAD and related metabolites Cerebra metabolism patterns Alzheimer’s disease assessment scale-Cognitive Subscale Clinical dementia rating—check sum of boxes MoCA changes Trail making test Lawton instrumental activities of daily living (IADL) scale Physical Self-Maintenance Scale (PSMS) Neuropsychiatric Inventory brief questionnaire form (NPI-Q) Montgomery-Asberg Depression Rating Scale (MADRS)	N/A
36.	NCT04044131	A phase 2, randomized, placebo-controlled study to evaluate the efficacy, tolerability, and safety of metabolic cofactor supplementation in Alzheimer’s disease (AD) and Parkinson’s disease (PD) patients	Istanbul Medipol University Hospital Antalya, Turkey Istanbul, Turkey	Age ≥ 18 years Diagnosis of AD or PD ADAS ≥ 12 CDR ≤ 2 No history of chronic illnesses No alcohol or substance abuse Normal TSH range No diabetes	Parallel assignment Experimental: Dietary supplementation with N-acetylcysteine, L-carnitine tartrate, nicotinamide riboside, and serine Placebo Comparator: 5 g Sorbitol	Randomized; quadruple blind	120	12 weeks	No results; completed Measures include: MMSE ADAS-cog Alzheimer’s Disease Cooperative Study-Activities of Daily Living (ADCS-ADL) Unified Parkinson’s Disease Rating Scale (UPDRS) MRI and rest-fMRI Neuropsychiatric inventory MoCA Serum omic profile Microbiota analysis Heart rate Blood Pressure Body weight CBC Liver function (ALT, AST, Albumin) Blood lipid levels (HDL, LDL, total cholesterol, total triglyceride) Kidney function Creatine kinase TSH Insulin Glycated hemoglobin Blood glucose	N/A
37.	NCT05546567#	NOPARK open label extension study	Haukeland University Hospital Bergen, Vestland, Norway	All ages All sexes No vitamin B3 supplementation	Single group assignment Experimental: 1200 mg Nicotinamide Riboside	N/A; open label	400	3 years	No results; recruiting Measures include: Safety and measurement of adverse events Total movement disorders society unified Parkinson’s disease rating scale (MDS-UPDRS) score. Range 0–199	N/A
38.	NCT03816020	NAD-PARK: a double-blinded randomized pilot trial of NAD-supplementation in drug naïve Parkinson’s disease	Haukeland University Hospital Bergen, Hordaland, Norway	Age ≥ 18 years Newly diagnosed idiopathic PD No dementia No metabolic, neoplastic, or other disorders	Parallel assignment Active comparator: 500 mg Nicotinamide Riboside BID Placebo comparator: placebo capsules BID	Randomized; triple blind	30	4 weeks	No results; completed Measures include: PDRP changes Motoric change of symptoms NAD metabolism	N/A
39.	NCT05589766#	N-DOSE: a dose optimization trial of nicotinamide riboside in Parkinson’s disease	Haukeland University Hospital Bergen, Vestland, Norway	Age: 40–100 years DAT-scan confirmation of nigrostriatal degeneration Hoehn and Yahr score < 4 No dementia No psychiatric illness No metabolic, neoplastic, or other illnesses No Vit. B3 supplementation	Parallel assignment Placebo comparator: Placebo tablet twice daily Experimental: 500 mg Nicotinamide Riboside (NR) twice a day Experimental: Dose escalation group: 1000 mg NR daily in doses of 500 mg twice daily (week 1–week 4), 2000 mg NR daily in doses of 1000 mg twice daily (week 5–week 8), 3000 mg NR daily in doses of 1500 mg twice daily (week 9–week 12)	Randomized; triple blind	80	12 weeks	No results; recruiting Measures include: Cerebral NAD levels CSF NAD and metabolite levels Expression of Nicotinamide Riboside Related Pattern (NRRP) International Parkinson and Movement Disorder Society Non-Motor Rating Scale (MDS-NMS) MoCA Gastrointestinal Dysfunction Scale (GIDS-PD) International Parkinson and Movement Disorder Society Unified Parkinson’s Disease Rating Scale (MDS-UPDRS)	N/A
40.	NCT03568968#	A randomized controlled trial of nicotinamide riboside supplementation in early Parkinson’s disease: the NOPARK Study	Haukeland University Hospital Arendal, Norway Bergen, Norway Drammen, Norway Førde, Norway	Age ≥ 35 years Diagnosis of idiopathic PD Hoehn and Yahr score < 3 No dementia No psychiatric disorder No use of Vit. B3 supplement No metabolic, neoplastic, or other illnesses No severe somatic illness	Parallel assignment Experimental: 500 mg nicotinamide riboside (NR) twice a day Placebo comparator: placebo capsule twice a day	Randomized; double bind	400	52 weeks	No results; recruiting Measures include: MDS-UPDRS Nigrostriatal degeneration by DaT scan Clinical severity of non-motor symptoms MoCA Euro quality of life five dimensions (EQ-5D-5L) questionnaire	N/A
41.	NCT05344404	NR-SAFE: a safety study investigating treatment with high-dose nicotinamide riboside (NR) in Parkinson’s disease	Haukeland University Hospital Bergen, Norway	Age ≥ 35 years Diagnosis of idiopathic PD Hoehn and Yahr score < 4 No dementia No psychiatric disorders No use of Vit B3 supplements No metabolic, neoplastic, or other illnesses No severe somatic illness	Parallel assignment Experimental: 1500 mg nicotinamide riboside twice per day Placebo comparator: placebo drug	Randomized; triple blind	20	4 weeks	No results; completed Measures include: Assessment of severe and non-severe adverse events NAD metabolome in blood and urine Clinical severity of pd (UPDRS)	N/A
		Niacin								
42.	NCT03462680	GPR109A and Parkinson’s Disease: Role of Niacin in Outcome Measures	VA Office of Research and Development Augusta, Georgia, United States	Age ≥ 35 years Diagnosis of PD MMSE < 24 No other chronic illness No dementia No allergies No functional blindness or lower limb amputation	Parallel assignment Active comparator: 250 mg niacin Placebo Comparator: Placebo tablet	Randomized; quadruple blind	47	6 months	UPDRS: not significant REM sleep pattern: not significant Deep sleep: not significant Light sleep: not significant MMSE: not significant CSF IL-6: not significant CSF IL-10: not significant	Not serious: 33.33% (flushing)

A clinical trial aimed at elucidating the effect of Insulin Sensitizer Metformin on AD Biomarkers (NCT01965756) used the Alzheimer’s Disease Assessment Scale-Cognitive Subscale (ADAS-Cog) to assess the potential of Metformin in mitigating AD-associated cognitive decline. The study also assessed the CSF concentration of Aβ, Tau, and pTau over a total period of 16 weeks. The ADAS-Cog test revealed no significant results. All clinical trials on the CRMs for aging and age-related neurodegenerative diseases are summarized in Table 3.

**Fig. 5:**
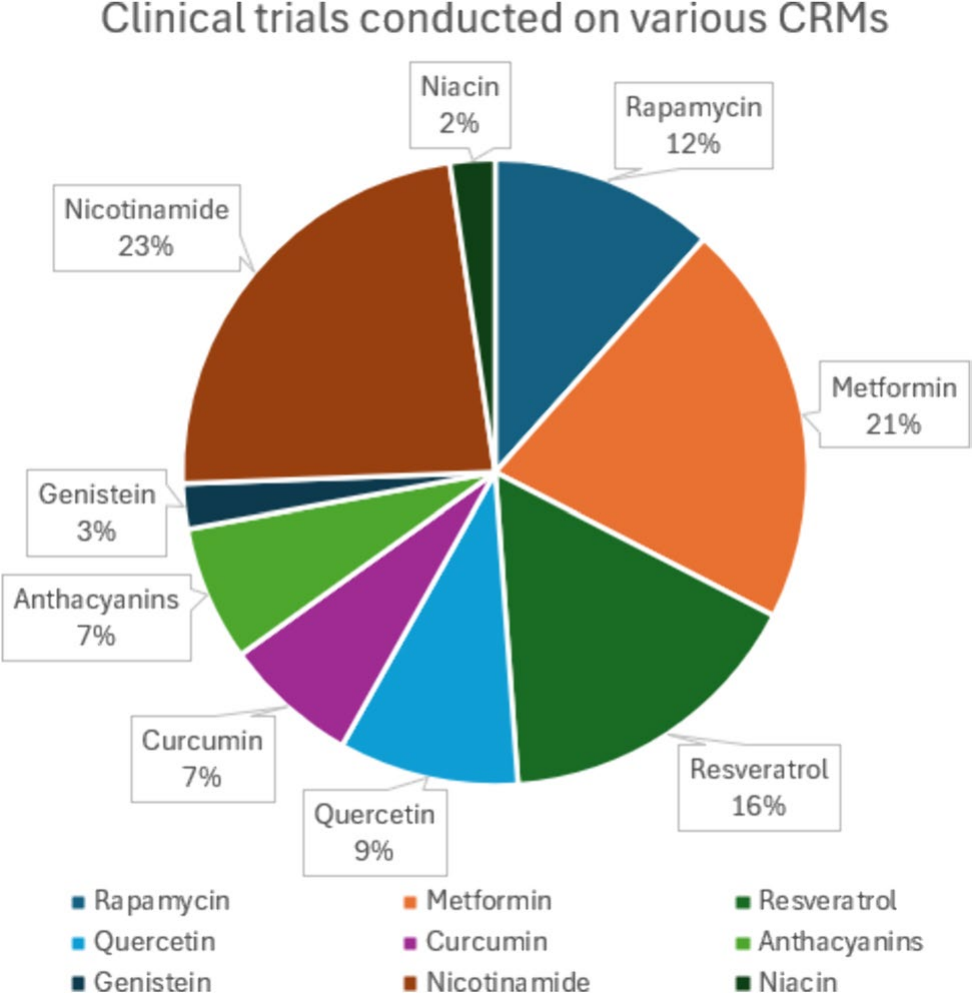
A pie chart depicting the percentage of clinical trials conducted on each CRM

Figure 5 outlines the proportion of trials that have been conducted on each CRM. According to this chart, most of the trials are centered around rapamycin (12%), metformin (21%), resveratrol (16%), and nicotinamide (23%). The lack of conclusive results despite many trials points towards the need for addressing the drawbacks and devising new strategies. Some limitations have been discussed in the subsequent section. Based on this view, trials need to be developed incorporating innovative solutions to yield beneficial results from these compounds. On the other hand, there is a dearth in the number of human trials conducted on curcumin, quercetin, genistein, niacin, and anthocyanins. This can be attributed to a lack of mechanistic understanding of these compounds, with no proper elucidation of molecular pathways and a wide array of target populations. This chart provides an estimate of the CRMs that are yet to be investigated, providing scope for clinicians and researchers to further design studies based on knowledge gaps and saturation percentage.

## Addressing the Limitations

With a lack of promising results, it is important to address the shortcomings of the completed trials. In any trial, certain parameters can be modulated to increase its efficiency. These include case ascertainment, target population, goal of therapy, dose selection, study duration, and confounders and interactions.

### Target Population

When recruiting for a case study, it is important to elucidate the inclusion and exclusion criteria in detail. However, such limited inclusions are also not ideal for translational properties amongst the public. The CALERIE study (Table 1, S. No. 1), as discussed in Sect. 4, has shown to be effective amongst the test subjects. However, the inclusion criteria are very specific to healthy-aged adults without any chronic illnesses and accompanying comorbidities. This limits the results of the study to a marginal population of the elderly and thus may have varying effects on aged individuals who suffer from common age-associated comorbidities like diabetes and hypertension (Vaiserman et al. 2020). Clinical trials on CR that are still recruiting like the OMIT study (Table 1, S. No. 3) also do not include aged individuals with common age-associated illnesses, and therefore this results in an incomplete assessment of the efficacy of the CR regimen. Moreover, potential side effects occurring by way of interactions between these comorbidities and the regimen may go undetected and cause unprecedented problems in its application. Therefore, future studies should incorporate populations with less severe illnesses to ascertain its effectiveness on a broader range of individuals (Nielsen et al. 2022).

On the other hand, when designing clinical trials related to NDDs, the inclusion criteria should be more stringent in including only those members of the population that show specific biomarkers of the disease (Babbs 2014). For AD, the onset of AI-based diagnostic tools has enhanced the specific detection of blood biomarkers that can be directly correlated with CSF concentrations of Aβ and Tau tangles (d’Abramo et al. 2020; Abukuri 2024; Cheng et al. 2024). More precise diagnostic measures should be undertaken to avoid the inclusion of participants who do not indeed suffer from AD. Clinical trials do not specify biomarker-based inclusion of participants and therefore individuals suffering from dementia unrelated to Alzheimer’s disease are also included in the study methodology. This can lead to a decline in the reported efficacy of the treatment as no changes in the outcome measures will be observed in such individuals (Huang et al. 2023). In the case of PD, a similar pattern is observed, with no specific inclusion criteria established for PD-related biomarkers (Gwinn et al. 2017). Lastly, for clinical trials that are inclusive of scoring-based questionnaires, individuals suffering from different ranges of cognitive impairment should be classified into different groups. This might allow for a more comprehensive analysis of the treatment on varying degrees of disease progression, allowing for the assessment of an optimal window of drug action (McCollum and Karlawish 2020).

It is important to realize that CR and CRMs are not effective in reversing disease progression. Rather, they are senolytic molecules that, over time, may confer neuroprotection and slow the onset of the disease (Gonzales et al. 2023). Trials need to be designed around participants diagnosed with early-onset AD or PD.

### Dose Selection

To optimize the clinical trial, it is necessary to conduct proper Phase 1 and Phase 2 clinical trials to ascertain the pharmacodynamic and pharmacokinetic properties of our molecule of interest before establishing other outcome measures (Alsultan et al. 2020). There is a lack of trials that establish an optimal dose at which the CRM in question shows beneficial effects. For instance, in the case of Rapamycin, the CARE trial (NCT01649960; Table 3, S. No.15) is a phase 1 trial that aims to establish the optimal dose at which SASP (interleukin 6, Matrix metalloproteinase 3, and Monocyte chemotactic protein 1) levels are at a minimum without incurring any serious adverse events. The trial is specific for aged individuals with no chronic illnesses. The study design follows a pattern wherein 3 individuals are assigned to a low dose. If one develops an adverse event, 3 more are assigned. Provided the three individuals enrolled did not show any side effects, the dosage will be increased, or else the trial will be terminated. This is an optimal strategy for determining the ideal dosage of the treatment. Similar regimens need to be established for trials recruiting AD and PD patients. Drug interactions may differ in healthy vs. diseased individuals due to interference caused by pathophysiological features of the disease as well as interactions between prescribed medication and the treatment drug (Cascorbi 2012). Therefore, the establishment of clear drug-drug interactions is a necessary stepping step before the advent of Phase 3 and Phase 4 trials.

### Outcome Measures

Several outcomes are routinely assessed for aging and most ongoing trials have made a good effort to include as many criteria as possible depending on the nature of the study. Due to the diverse consequences of aging, it is not possible to encompass all measures in a single trial, instead, trials have to be forthcoming to target a specific aspect of aging and enumerate the effect of the treatment on the same (Syed et al. 2022). For instance, the PEARL study for rapamycin (NCT04488601; Table 3, S. No. 11) includes a series of measures on blood composition, hemoglobin acetylation levels, bone density, insulin and cholesterol profile, and other parameters indicative of kidney functioning including enzymatic levels of alkaline phosphatase and bilirubin. The study clearly outlines its endpoints but does not do a detailed analysis of either one of the aging parameters. On the other hand, a study (NCT02123121; Table 3, S. No. 19) on resveratrol’s effects on mitochondrial functioning clearly outlines specific parameters that translate to mitochondrial efficiency like COX gene expression, citrate synthase levels, changes in mtDNA, and activation of AMPK and sirtuins. This study design is a good example of introducing specificity in the outcome measures and elucidating the effects of the treatment on one aspect of the condition. There are very few trials that include measures of gene expression of the important cellular pathways associated with CRMs. The inclusivity rate of molecular expression parameters for mTOR and sirtuins should be increased. In the case of PD, all outcome measures are based on subjective scales that rank the extent of disease progression. There is a dearth of blood-based biomarkers that are assessed for determining the efficacy of the treatment against PD.

### Pharmacological Properties of the Drug

While certain compounds show beneficial effects in vivo and in vitro, it is necessary to evaluate the efficiency of these compounds and the discrepancies in their pharmacokinetic and pharmacodynamic properties when translating from animal models to humans (Yadav et al. 2021). In general, the translational merit of drugs has been low with an overall low correlation value when tested for roughly 184 drugs. These discrepancies arise as a result of differences in enzymatic metabolism, physiological processes, and variations in drug formulation (direct injection, tablets, or syrups) (Lai et al. 2022). A matter of concern is the bioavailability of molecules in specific tissues. All CRMs show relatively low bioavailability except NAD^+^ precursors, nicotinamide, and niacin, which show high absorption and are water-soluble allowing their appropriate distribution (She et al. 2021). Most CRMs, including Resveratrol, EGCG, Apigenin, Quercetin, Curcumin, Astragalin, and others display rapid metabolism into their respective metabolites. Some molecules undergo first-pass metabolism which significantly reduces the quantity of active molecules to circulate in the tissues (Shabbir et al. 2021). Some molecules display higher efficiencies when conjugated with other dietary elements. For instance, anthocyanins display an enhanced bioavailability in the presence of dietary fats (Ayvaz et al. 2022) whereas curcumin formulations with piperine (black pepper) can show similar effects (Prasad et al. 2014). The low bioavailability of compounds can be tackled by formulating novel ways of drug delivery including nanoparticle formulation, which in the case of resveratrol has shown improved effects (Kumari et al. 2022). Additionally, methods to reconvert the metabolites into active molecules within the target molecules should be explored for their potential beneficial effects. Another approach is to characterize the effect of the metabolites themselves or adopt techniques of entero-hepatic recycling (Tu et al. 2021).

## Future Directions

Future studies should further expand the target populations so that typical age-associated comorbidities can be addressed in the trials. This would help in understanding how CR and CRMs work with the different patient groups that have diseases such as diabetes and hypertension. Moreover, embedding biomarker-based criteria of neurodegenerative diseases, like AD and PD, will enhance specificity in participant selection and ensure that only subjects with relevant pathologies are enrolled. This may enable a more accurate assessment of treatment efficacy and safety during clinical trials. Full development of optimal dosing regimens based on well-structured Phase 1 and Phase 2 trials will be important. Studies in the future should delve into the pharmacodynamics and pharmacokinetics of CRMs in identifying effective doses with the least number of adverse events. Most importantly, co-prescription should be studied in populations with pre-existing conditions to facilitate safety and effectiveness at the real-world level. Clinical trials targeting specific parameters of aging or disease progression, with state-of-the-art biomarkers and molecular expression parameters relevant to the CRMs under test, should now be conducted. Such specificity will allow much clearer insight into the effects of treatments and thus raise the translational potential of results. Future studies should also be directed at the low bioavailability of many CRMs by the development of innovative drug delivery systems that could include nanoparticle formulations. Further pharmacokinetic evaluations of the metabolites and their potential therapeutic value might provide such insights, which could contribute to further improving the overall effectiveness of CRMs. These are the processes that should be tested to obtain the highest pharmacological activity of such compounds.

Addressing these areas in future clinical trials could therefore go a long way toward advancing the understanding and application of CR and CRMs in the promotion of health for better aging and better control of diseases associated with aging.

## Conclusion

Age-associated cognitive decline is an inevitable phenomenon. In today’s world of sedentary lifestyles, there is now a pressing need to introduce naturally derived compounds like CRMs that can mimic the effects of a healthy lifestyle without disrupting other bodily systems. CRMs have been in the limelight for their therapeutic potential for over a decade. The mechanisms underlying their modes of action, although not completely understood, have been sufficiently elucidated to devise specific trials measuring biomarkers of aging and age-associated NDDs. CRMs have become popular candidates in clinical settings due to their accessibility and lack of very serious side effects. All clinical trials on various interventional models were retrieved from the clinicaltrials.gov database. Some studies displayed their results while some, although completed, have not posted any results on the database. Some studies were terminated due to lack of funding or insufficiency of participants. Despite the abundance of clinical trials conducted on CRMs, there is a dearth of significant results that carefully corroborate the efficacy of CRMs against aging and age-related neurodegenerative disorders. A summary of the clinical trials reflected very few adverse events upon CRM administration with most studies listing side effects caused due to other factors. However, no clinical trials have been able to produce significant results. This may be attributed to the short nature of the trials. The CALERIE study, which received an enthusiastic response for its positive result, was conducted for over 2 years whereas the other studies have been investigated for shorter durations. This highlights the necessity for long-term interventional studies that can assess the prolonged effect of CRMs on longevity and well-being. Moreover, combined therapies alongside exercise and conventional medicine should be explored to determine whether CRMs have an additive effect on already existing therapeutic agents.

## Data Availability

No datasets were generated or analysed during the current study.

## Abbreviations

NDD: Neurodegenerative disorder
CR: Caloric restriction
mTOR: Target of rapamycin
CRM: Caloric restriction mimetics
ROS: Reactive oxygen species
IL-6: Interleukin-6
TNF-α: Tumor necrosis factor-alpha
IL-1β: Interleukin 1 beta
AD: Alzheimer’s disease
PD: Parkinson’s disease
NFTs: Neurofibrillary tangles
APP: Amyloid precursor protein
BACE1: β-Secretase 1
AChE: Acetylcholinesterase
DA: Dopamine
mTOR: Mammalian target of rapamycin
AMPK: 5′-Adenosine monophosphate-activated protein kinase
ADAS-Cog: Alzheimer’s disease assessment scale- cognitive subscale
CSF: Cerebrospinal fluid
Aβ: Amyloid beta
pTau: Phosphorylated tau
SASP: Senescence-associated secretory phenotype
mtDNA: Mitochondrial DNA
BMI: Body mass index
IUD: Intra-uterine device
CVD: Cardiovascular disease
UTI: Urinary tract infection
BMAL1: Brain and muscle ARNT-like 1
Cry1: Cryptochrome 1
Per2: Period circadian regulator 2
Nr1d1: Nuclear receptor subfamily 1 group d member 1
Nfil2: D-site of albumin promoter (albumin d-box) binding protein
SOD2: Manganese superoxide dismutase
GPx1: Glutathione peroxidase 1
SIRT1: Sirtuin 1
SIRT3: Sirtuin 3
NRF1: Nuclear Respiratory Factor 1
8oxodG: 8-Hydroxy-2′-deoxyguanosine
TRE: Trans-regulatory elements
MoCA: Montreal cognitive assessment
SPPB: Short physical performance battery
HbA1C: Glycated hemoglobin
STICSA: State-trait inventory for cognitive and somatic anxiety
LDL: Low-density lipoprotein
HDL: High-density lipoprotein
NDEV: Neuronal-derived extracellular vesicle
CDR: Complementarity-determining region
MMSE: Mini-mental state examination
SF-36: Short form (36) health survey
IGF-1: Insulin-like growth factor 1
cAMP: Cyclic amp
PKA: Protein kinase A
COX: Cyclooxygenase
ERK: Extracellular regulated kinase
BDNF: Brain derived neurotrophic factor
NF-κB: Nuclear factor kappa-light-chain-enhancer of activated B cells
PI3K: Phosphatidylinositol 3-kinase
Atg: Autophagy-related genes
MCI: Mild cognitive impairment
FCSRT: Free and cued selective reminding test
UPDRS: Unified Parkinson’s disease rating scale
BP: Blood pressure
GSH: Glutathione
